# Dual-scale chemical ordering for cryogenic properties in CoNiV-based alloys

**DOI:** 10.1038/s41586-025-09458-1

**Published:** 2025-08-27

**Authors:** Tiwen Lu, Binhan Sun, Yue Li, Sheng Dai, Ning Yao, Wenbo Li, Xizhen Dong, Xiyu Chen, Jiacheng Niu, Fan Ye, Alisson Kwiatkowski da Silva, Shuya Zhu, Yu Xie, Xiaofeng Yang, Sihao Deng, Jianping Tan, Zhiming Li, Dirk Ponge, Lunhua He, Xian-Cheng Zhang, Dierk Raabe, Shan-Tung Tu

**Affiliations:** 1 Key Laboratory of Pressure Systems and Safety, Ministry of Education, East China University of Science and Technology, Shanghai, China; 2 Max Planck Institute for Sustainable Materials, Düsseldorf, Germany; 3 Key Laboratory for Advanced Materials and Joint International Research Laboratory of Precision Chemistry and Molecular Engineering, Feringa Nobel Prize Scientist Joint Research Centre, School of Chemistry and Molecular Engineering, East China University of Science and Technology, Shanghai, China; 4 National Engineering Research Center of Near-net-shape Forming for Metallic Materials, South China University of Technology, Guangzhou, China; 5 School of Materials Science and Engineering, Central South University, Changsha, China; 6 Spallation Neutron Source Science Center, Dongguan, China; 7 Beijing National Laboratory for Condensed Matter Physics, Institute of Physics, Chinese Academy of Sciences, Beijing, China

**Keywords:** Metals and alloys, Mechanical properties, Mechanical engineering

## Abstract

The mechanical properties of metallic materials often degrade under harsh cryogenic conditions, posing challenges for low-temperature infrastructures^1^. Here we introduce a dual-scale atomic-ordering nanostructure, characterized by an exceptionally high number density of co-existing subnanoscale short-range ordering (approximately 2.4 × 10^26^ m^−3^) and nanoscale long-range ordering (approximately 4.5 × 10^25^ m^−3^) domains, within a metallic solid-solution matrix in a CoNiV-based alloy to improve the synergy of strength and ductility at low temperatures. We observe an ordering-induced increase in dislocation shear stress as well as a more rapid dislocation multiplication owing to the dislocation blocking effect of nanoscale long-range ordering and the associated generation of new dislocations. The latter effect also releases stress concentrations at nanoscale long-range-ordered obstacles that otherwise would promote damage initiation and failure. Consequently, the alloy shows a strength–elongation product of 76 GPa % with a yield strength of approximately 1.2 GPa at 87 K, outperforming materials devoid of such ordering hierarchy, containing only short-range ordered or coherent precipitates of a few tens of nanometres. Our results highlight the impact of dual co-existing chemical ordering on the mechanical properties of complex alloys and offer guidelines to control these ordering states to enhance their mechanical performance for cryogenic applications.

## Abstract (web summary)

Dual-scale chemical ordering in CoNiV-based alloys improves the synergy of strength and ductility at cryogenic temperatures, providing an approach for obtaining high-performance metallic materials for cryogenic applications.

## Main

Most alloys and their microstructures have been developed over recent decades to provide the desired mechanical performance at room and elevated temperatures. However, fewer material options exist for high-strength and damage-tolerant cryogenic applications, despite the less severe cost restrictions in this field^2,3^. For instance, precipitation hardening has been one of the most successful strategies to enhance the strength of alloys, by impeding dislocation motion, which leads to higher loads for maintaining inelastic deformation^4–8^. However, under cryogenic conditions, this approach comes at the expense of ductility, owing to formation of dislocation pile-ups and high stress concentrations at the precipitate–matrix interfaces, both features that promote damage initiation at these sites^9,10^. The formation of coherent precipitates (for example, the L1_2_ long-range-ordered (LRO) phase in some face-centred cubic (fcc) materials) can to some extent reduce the damage initiation tendency owing to the lower interfacial energy compared with that for incoherent and semi-coherent interfaces^11^. However, an intense heterogeneous stress field can still develop when the size of these precipitates becomes sufficiently large after prolonged ageing, owing to a high anti-phase boundary energy and the formation of dislocation loops around them impeding the ensuing dislocation motion^9,12,13^.

In contrast to these well-established hardening mechanisms and their inherent disadvantages that are known from conventional dilute alloys, the impact of subnanoscale chemical short-range ordered (SRO) features on mechanical properties has been less understood^14–16^. These atomic ordering clusters seem to form in some medium- and high-entropy alloys (MEAs and HEAs) as a result of a subtle interplay between local enthalpic interactions among the constituent elements and the otherwise entropy-dominated solid solution on which these alloys are based^14^. The highly dispersed and uniformly distributed SRO zones, along with their suppressive effect on easy dislocation glide and the associated low stress and strain heterogeneity^16–18^, turn out to be an ideal strengthening mechanism at cryogenic temperatures, although the underlying strengthening mechanisms and their tunability have remained elusive so far^14–16^. However, with respect to the influence on strain-hardening capacity (ensuring uniform elongation), SRO domains seem to be less effective than LRO domains^15,19^. This discrepancy between appreciable stress increase and insufficient strain hardening motivates us to develop a rationale design concept for dual-scale chemical ordering. In this context, we show that a fine balancing of two types of nanoscale ordered structures and a massive solid-solution matrix design can become a key approach for the development of high-performance metallic materials for cryogenic applications.

**Fig. 1:**
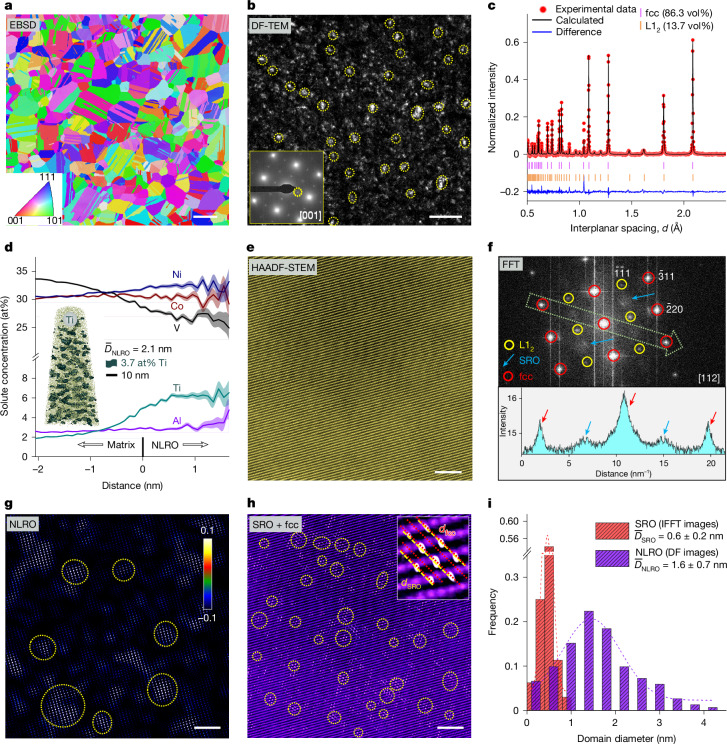
Microstructure and dual-scale chemical ordering of our CoNiV-AlTi sample. **a**, Electron backscatter diffraction (EBSD) inverse pole figure showing the fcc matrix with an average grain size of 11.2 ± 1.3 μm. **b**, DF-TEM image showing the presence of NLRO zones. The inset is the selected-area electron diffraction (SAED) pattern taken from the [001] zone axis, and the DF-TEM image was taken from one of the superlattice spots marked by the yellow dotted circle in the SAED pattern. **c**, Neutron diffraction pattern showing the fcc and L1_2_ peaks. The calculated weight fraction of L1_2_ phase was converted to the volume fraction. **d**, APT proximity histogram concentration profile across the matrix–NLRO interface based on a 3.7 at% Ti iso-concentration surface. The shaded regions are error bars corresponding to their coloured lines (element distribution). Inset: the iso-concentration surface map for Ti. **e**,**f**, Atomic-scale HAADF-STEM image along the [112] zone axis (**e**) and the corresponding FFT pattern collected from one fcc grain (**f**). Inset in **f**: the intensity profile along the light green arrow marked in the FFT pattern. **g**,**h**, IFFT image transformed from the NLRO (**g**) and SRO (**h**) spots in the FFT pattern in **f**. Some typical NLRO and SRO domains are marked by yellow dotted circles. Inset in **g**: scale bar for the normalized NLRO intensity. Inset in **h**: an enlarged region showing the detailed lattice structure of a SRO domain and the fcc matrix. **i**, Distribution of the size of NLRO and SRO domains, which was statistically analysed based on DF-TEM images and IFFT images, respectively. Scale bars, 10 μm (**a**), 20 nm (**b**), 2 nm (**e**,**g**,**h**).

**Extended Data Fig. 1:**
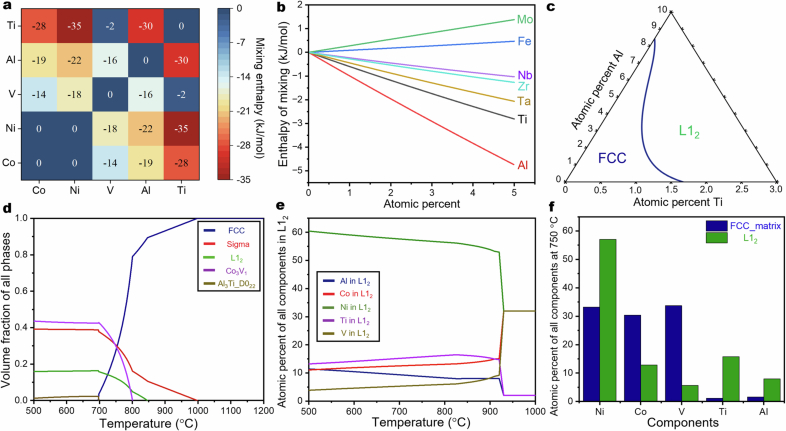
Thermodynamic evaluation for the ordering structure in the Co_32_Ni_32_V_32_Al_2_Ti_2_ alloy. (a) Enthalpy of mixing among the elements involved in our materials. (b) Enthalpy of mixing of different elements in the fcc phase with CoNiV composition (equimolar) at 1100 °C. Al and Ti have the most negative enthalpy of mixing with the main elements, which strongly enhances the tendency for the formation of long-range ordering. (c) The promoting effect of Al and Ti addition on the formation of the L1_2_ ordered phase aged at 750 °C, assuming that the main elements have a same atomic concentration (N(Ni) = N(Co) = N(V)). (d) Equilibrium volume fraction of different phases as a function of temperature. (e) Equilibrium composition of the L1_2_ precipitates predicted by Thermo-Calc software. (f) Equilibrium composition (at.%) of fcc_matrix and L1_2_ phases aged at 750 °C.

We realize this nanostructure design strategy in a CoNiV-based alloy, with a nominal composition of Co_32_Ni_32_V_32_Al_2_Ti_2_ (at%), hereafter referred to as CoNiV-AlTi. The enthalpic interactions of vanadium (V) with cobalt (Co) and nickel (Ni), as well as their significant mismatch in atomic size and shear modulus (Supplementary Table 1), promote the formation of local chemical short-range ordering^17,20^. Among the frequently added microalloying elements, aluminium (Al) and titanium (Ti) have the most negative enthalpy of mixing with the main elements (Extended Data Fig. 1), which strongly enhances the tendency for the formation of long-range ordering^21,22^. A careful thermodynamically guided solid-solution and ageing treatment is next required to bring these two types of enthalpy-dominated ordering state into co-existence with the entropy-dominated massive solid-solution matrix, without transitioning into a state of intermetallic or larger precipitate formation (Supplementary Fig. 1). The so-treated alloy has a recrystallized and chemically homogeneous fcc matrix (Fig. 1a and Supplementary Fig. 2) with a high number density (approximately 4.5 × 10^25^ m^−3^) of nanoscale L1_2_ long-range ordered (NLRO) domains, as shown in the dark-field transmission electron microscopy (DF-TEM) image (Fig. 1b). The average size of the NLRO domains is 1.6 ± 0.7 nm, which is at least half an order of magnitude smaller than L1_2_-structured precipitates often observed in some MEAs and HEAs or high-strength steels (normally above 5 nm)^22–24^. The fraction of the nanoscale L1_2_ domains occupies 13.7 vol%, as determined from neutron diffraction experiments and Rietveld refinement analysis (Fig. 1c). Atom probe tomography (APT) analysis reveals an enrichment of Ti and Ni in this domain (Fig. 1d). On the basis of the thermodynamic calculation (Extended Data Fig. 1), these NLRO domains are the early-stage precursor states of close-packed Ni_3_(Ti, Al)-type L1_2_ precipitates that are typically formed by spinodal decomposition. The determined average Ti content of the NLRO domain (6.2 at%) is lower than that predicted by thermodynamic calculations (15.7 at%, according to the TCHEA4 database and the Thermo-Calc software), owing to the limited ageing time applied in our case, that is, equilibrium partitioning by purpose has not been obtained, to reach atomic-scale ordering without transition into the full equilibrium precipitation state. Such restricted compositional contrast between the NLRO domains and the matrix yields an ultralow lattice mismatch of 0.04% between the two phases, as revealed by neutron diffraction.

**Extended Data Fig. 2:**
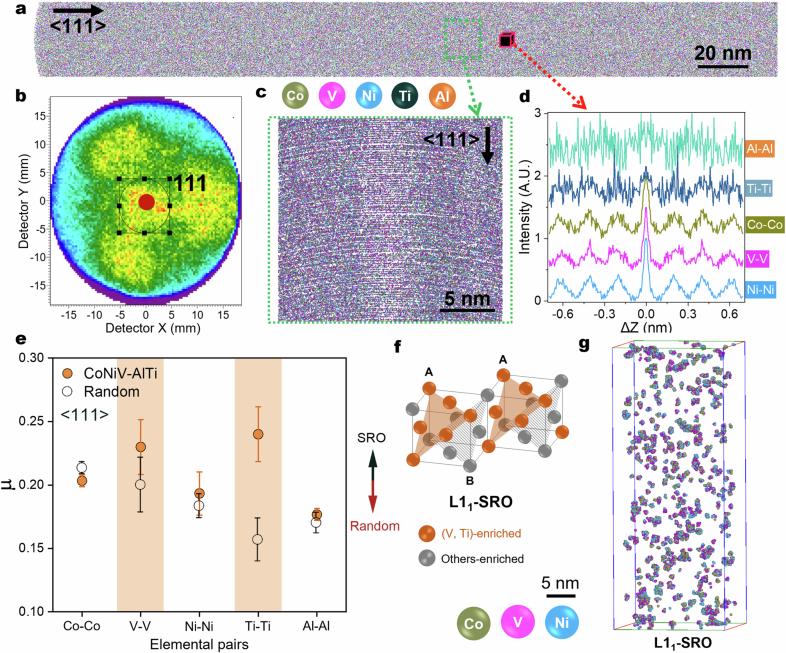
APT data and derived L1_1_-typed chemical SRO configuration in the CoNiV-AlTi alloy along the <111> grain orientation. (a) Precise 3D APT reconstruction along the <111> grain orientation. (b) Representative 2D detector hit map. One centric {111} crystallographic pole is labeled. (c) A close-up of a thin slice along <111 > . (d) The spatial distribution maps (z-SDMs) of Co-Co, Ni-Ni, V-V, Al-Al and Ti-Ti pairs in a 2 × 2 × 2 nm^3^ voxel in a. (e) Pearson contingency coefficient (µ) of each elemental pair along the <111> grain orientation. The faint yellow regions highlight the main changes of µ departing from the random state. Note that different from the equiatomic ternary system, it is necessary to define separate threshold values for each elemental pair in non-equiatomic systems due to the significantly different size distributions observed in the randomized datasets. (f) Deduced structure and elemental occupancy in L1_1_-SRO. (g) Representative 3D distributions of recognized L1_1_-SRO. More details of this method can be referred to Supplementary Note 3. Here the error bars represent the standard deviations.

Figure 1e shows a representative atomic-resolution high-angle annular dark-field scanning transmission electron microscopy (HAADF-STEM) image of the alloy, taken along the [112] zone axis, with the corresponding fast Fourier transform (FFT) pattern shown in Fig. 1f. In addition to the sharply concentrated spots in the FFT image that represent the presence of the L1_2_ structure (marked by yellow circles in Fig. 1f), we also observe bright diffuse reflections, which locate at positions corresponding to $1/2\{\bar{3}11\}$ (indicated by the blue arrows in Fig. 1f). Such diffuse reflections, combined with machine-learning-enhanced APT (ML-APT) analysis (Extended Data Fig. 2), represent the presence of the L1_1_-structure SRO, which mainly originates from the nearest-neighbour preference towards unlike element pairs (such as V–Co or V–Ni pairs^17^). The inverse FFT (IFFT) images transformed from the L1_2_ and SRO reflections provide the size and distribution of these two classes of ordered structures (Fig. 1g,h), indeed revealing the co-existence of both types of nanoscale ordering structure features in one local region of an fcc grain. Compared with NLRO, the size of the SRO domains is much smaller, namely, about 0.6 ± 0.2 nm (Fig. 1i), thus assuming atomic-scale dimensions, and more densely distributed (number density about 2.4 × 10^26^ m^−3^). The existence of a high density of these ordering domains effectively reduces the dislocation mean free path, therefore increasing the critical resolved shear stress for dislocation gliding (Supplementary Note 1). This pushes the limits of rational thermodynamic nanostructure design of complex alloys into atomic-scale regions.

**Fig. 2:**
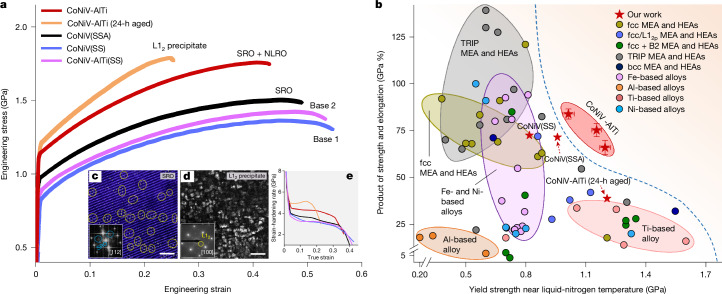
Cryogenic tensile property of the investigated alloys at 87 K. **a**, Engineering stress–strain curve of our CoNiV-AlTi sample with a dual-scale ordering structure, compared with the base CoNiV(SS) and CoNiV-AlTi(SS) samples, the CoNiV(SSA) sample, and the aged CoNiV-AlTi sample containing large-sized (about 25 nm) L1_2_ precipitates. **c**, IFFT image of the CoNiV(SSA) sample, showing the presence of SRO and its size and distribution. **d**, DF-TEM image of the 24-h aged CoNiV-AlTi sample, showing the presence of large-sized L1_2_ precipitates. **e**, Strain-hardening-rate curves. The grain size of all these materials is similar (Supplementary Fig. 5). **b**, The product of tensile strength and total elongation as a function of yield strength of the CoNiV-AlTi alloy (at 87 K) with different grain sizes (Supplementary Fig. 6), compared with other high-performance alloys used or planned to be used in cryogenic applications, including various MEA and HEAs (for example, single-phase fcc MEA/HEAs, fcc MEA/HEAs with L1_2_ or B2 ordered phases, transformation-induced plasticity (TRIP) MEA/HEAs, and body-centered cubic (bcc) MEA/HEAs), Fe-based alloys, Ni-based alloys, Al-based alloys and Ti-based alloys. All the data in this figure are from samples tested at near liquid-nitrogen temperature (77–110 K), and the associated details are listed in Supplementary Table 3. The error bars represent standard deviation. Scale bars, 1 nm (**c**), 50 nm (**d**).

**Extended Data Fig. 3:**
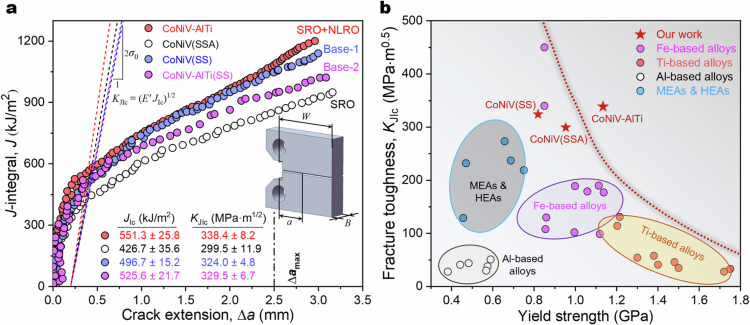
Cryogenic fracture toughness of the investigated alloys at 87 K. (a) Fracture toughness results of the CoNiV(SS), CoNiV(SSA), CoNiV-AlTi(SS) and CoNiV-AlTi samples at 87 K, showing an impressive fracture toughness of the CoNiV-AlTi sample with dual-scale ordering. The thickness (*B*) of C(T) specimens was 5.5 mm, and more details of fracture toughness tests and the associated calculation can be found in Supplementary Note 4. (b) An Ashby map of fracture toughness versus yield strength of different materials at cryogenic temperature (77 K–123 K). The CoNiV-AlTi sample shows better combination of yield strength and fracture toughness than Al-based alloys, Fe-based alloys, Ti-based alloys and medium/high-entropy alloys. More details of the literature data are listed in Supplementary Table 5. Here the error bars represent the standard deviations.

The introduction of dual-scale local chemical ordering and their careful size control result in an exceptional strength (yield strength about 1.2 GPa, ultimate tensile strength about 1.8 GPa) and ductility (fracture strain about 42.6 %) in our CoNiV-AlTi alloy at cryogenic temperature (87 K), as shown in Fig. 2a. Figure 2b reveals that at the same yield strength level, the product of strength and ductility of our alloy has surpassed corresponding reference values of other metallic materials (by about 60%) that have already been used or have potential to be used in cryogenic applications, including various HEAs and MEAs and more conventional Ti-based, Ni-based, Fe-based and Al-based alloys. A notably high fracture toughness (338.4 MPa m^0.5^) is also achieved at 87 K using our dual-scale ordering strategy (Extended Data Fig. 3).

**Extended Data Fig. 4:**
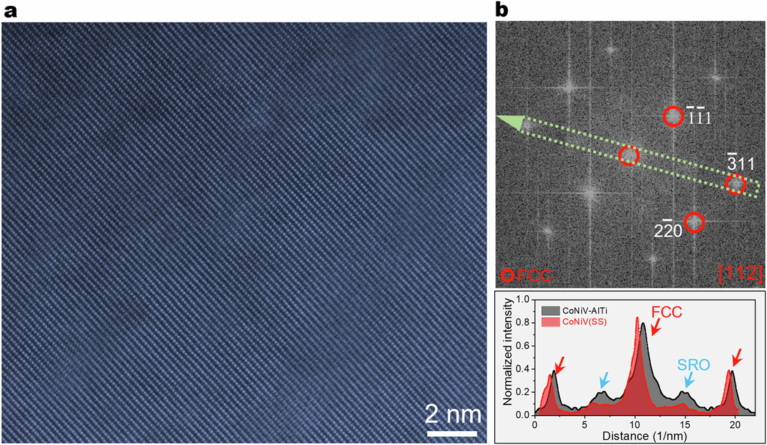
The ordering characterization of the solid-solution treated CoNiV(SS) alloy. (a) Atomic-scale HAADF-STEM image along the [112] zone axis. (b) The corresponding FFT pattern, with the negligible extra discs locate at positions corresponding to $1/2\{\bar{3}11\}$ with the negligible extra discs located at positions corresponding to $1/2\{\bar{3}11\}$. Normalized diffraction data along the light green dash lines is plotted in the down panel. In contrast to the obvious SRO sign in the CoNiV-AlTi sample, the diffraction signal of SRO in the CoNiV(SS) sample is rather weak.

**Extended Data Fig. 5:**
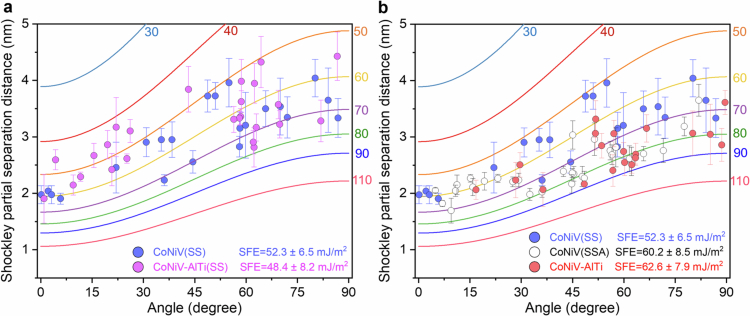
Detailed *g*·*b* analysis of partial dislocations in the cryogenically (87 K) strained (6%) samples and the calculation of SFE. (a) Measured dissociated distances as a function of the angle between the Burgers vector and dislocation line in solid-solution treated and cryogenically strained CoNiV(SS) and CoNiV-AlTi(SS) samples, which indicates that the addition of 2 at.% Al and 2 at.% Ti in our work has no significant influence on SFE. (b) The comparison among CoNiV(SS), CoNiV(SSA) and CoNiV-AlTi samples, revealing the role of ordering on SFE. Despite an increase role of SRO on CoNiV alloys’ SFE, the increment here (~8 mJ/m^2^) is not significant considering the measurement error (~8.5 mJ/m^2^). The detailed calculation procedure of SFE was presented in Supplementary Note 2.

**Extended Data Fig. 6:**
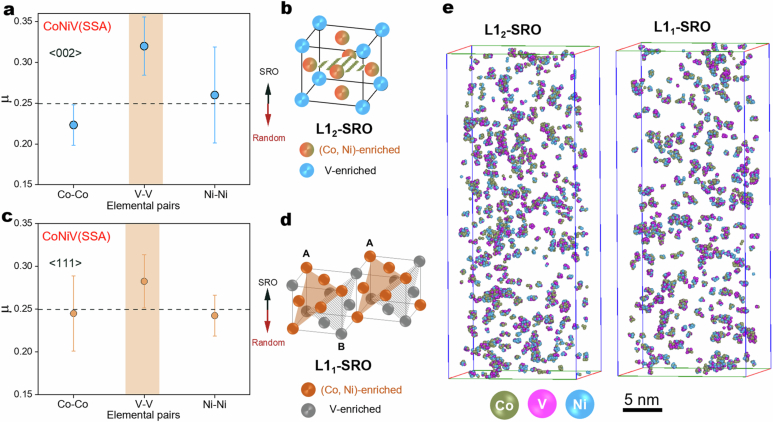
MP-APT analysis of aged CoNiV(SSA) sample. (a) and (c) Distributions of Pearson contingency coefficient (µ) along the <002> and <111> grain orientation, respectively. A value of µ=0.25 is regarded as the threshold between chemical SRO and random states. (b) The deduced L1_2_-SRO structure with the V-V repulsion along <002>. (d) The deduced L1_1_-SRO structure with the V-V repulsion along <111>. (e) Elemental mappings showing the 3D distributions of L1_2_- and L1_1_-SROs. Here the error bars represent the standard deviations. More details of this method can be referred to Supplementary Note 3.

To investigate the respective contribution of the SRO and NLRO domains on the cryogenic mechanical properties, we further compare our alloy with (1) an equimolar CoNiV base-alloy that is solid-solution treated (hereafter referred to as CoNiV(SS)), (2) a likewise solid-solution-treated CoNiV-AlTi sample (hereafter referred to as CoNiV-AlTi(SS)), and (3) a solid-solution-treated and aged CoNiV sample (hereafter referred to as CoNiV(SSA)). The first two types of material, directly quenched from high temperature (1,100 °C) at which the entropy-driven configurational intermixing exceeds the enthalpy-driven ordering effects^14^, contain a low possibility of SRO-domain formation (see the TEM results in Extended Data Fig. 4). We note that Al and Ti in solid solution (about 2 at% for each element) only slightly increase the yield strength (by 27 MPa) under cryogenic temperature loading conditions (Fig. 2a), but do not significantly alter the materials’ stacking fault energy (SFE) or their elastic properties (Extended Data Fig. 5 and Supplementary Table 2). Both, the CoNiV(SS) and CoNiV-AlTi(SS) samples are thus regarded as ordering-free base materials for comparison. The CoNiV(SSA) sample, aged at 750 °C, contains only SRO domains with a similar domain size and volume fraction as that in our alloy (Fig. 2c). Further thermodynamic assessment and ML-APT analysis, as shown in Supplementary Fig. 3 and Extended Data Fig. 6, also confirm the likelihood of the formation of SRO domains with both L1_1_ and L1_2_ atomic configurations in the CoNiV(SSA) sample.

**Extended Data Fig. 7:**
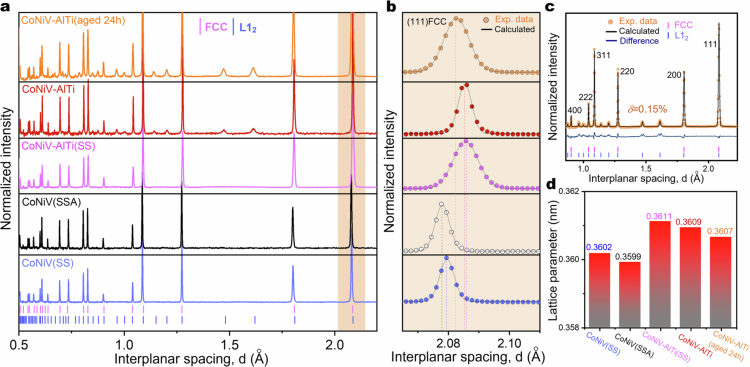
Neutron diffraction analysis of studied materials. (a) Neutron diffraction patterns of the CoNiV(SS), CoNiV(SSA), CoNiV-AlTi(SS), CoNiV-AlTi and CoNiV-AlTi (aged 24 h) samples before deformation. (b) Enlarged (111) peaks. (c) Neutron diffraction pattern and fitted curves of CoNiV-AlTi (aged 24 h) samples. When the LRO size increases from 1.6 nm to 25 nm, the lattice mismatch (*δ*) between LRO and the matrix increases from 0.04% for CoNiV-AlTi to 0.15% for CoNiV-AlTi (aged 24 h). (d) The lattice parameters of the investigated alloys were determined by the Rietveld method using a GSAS II software. There is a distinct difference in the lattice parameter of the FCC matrix between solid-solution treated and solid-solution treated plus aged samples, indicating the difference in the ordering degree. The lattice parameters of CoNiV(SS) and CoNiV(SSA) were determined to be 0.3602 nm and 0.3599 nm, respectively, indicating the difference in the degree of SRO between two samples, and the L1_2_ peaks were absent in two samples.

The comparison between the CoNiV(SS) and CoNiV(SSA) specimens reveals that the presence of SRO markedly increases the cryogenic yield strength (by about 140 MPa) but does not affect the alloy’s strain-hardening ability (Fig. 2e). Such an increase in yield strength is attributed to the additional energy barrier associated with the creation of diffuse anti-phase boundaries when dislocations pass through such SRO regions^16,25^. When NLRO domains are also introduced into the material (that is, our CoNiV-AlTi sample), the NLRO-induced enhancement in yield strength is about 167 MPa, with a similar strengthening mechanism as that from SRO domains^18,26^. Moreover, the strain-hardening rate is simultaneously elevated (by about 1,000 MPa) owing to the presence of NLRO domains, a key factor for the sustained ductility at cryogenic temperature. The introduction of the (sub)nanoscale ordering structure does not result in a significant ductility loss at cryogenic temperature exposure, which is in sharp contrast to a sample that is aged for prolonged time (24 h) to form larger ordered L1_2_ precipitates with an average size of about 25 nm (Fig. 2d). For the latter sample, the increased lattice misfit (0.15%; Extended Data Fig. 7) between the L1_2_ precipitates and the matrix, resulting from the larger precipitate size and the higher degree of compositional contrast, leads to an increase in the interfacial energy^27^ as well as in stress concentrations at the precipitate–matrix interfaces^9,12,13^. These factors promote early crack nucleation, particularly at cryogenic temperatures (Supplementary Fig. 4), which results in a significantly reduced ductility of this sample (only about 22%; Fig. 2a).

**Fig. 3:**
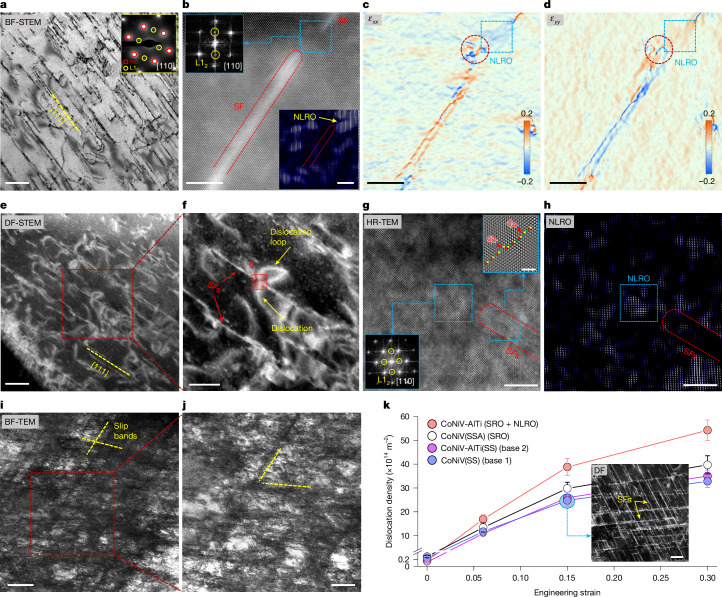
Typical deformation substructure of the CoNiV-AlTi alloy. **a**–**j**, Images of the substructure of the CoNiV-AlTi alloy cryogenically (87 K) deformed at 6% (**a**–**d**), 15% (**e**–**h**) and 30% (**i**,**j**) strain. **a**, Bright-field (BF) STEM image showing the formation of SFs along {111} slip planes. Top-right inset in **a**: the corresponding SAED pattern. **b**, Atomic-scale low-angle annular dark-field STEM image, the corresponding FFT pattern and the IFFT image, showing the existence of non-destructed NLRO domain in-between two SFs. Bottom-right inset in **b**: the IFFT image showing the distribution of NLRO. Top-left inset in **b**: the FFT pattern from the corresponding light blue dashed box.** c**,**d**, The distribution of elastic strain in horizontal (*ε*_*xx*_; **c**) and vertical (*ε*_*yy*_; **d**) directions for the same area in **b**, revealing the interaction between the NLRO domains and the SFs. The red dashed circle highlights the high elastic strain concentration close to a NLRO zone that is marked by a blue dashed square. **e**, DF-STEM image showing non-planar dislocations and dislocation loops between slip bands. **f**, The enlarged figure from the corresponding red dashed box in **e**. **g**,**h**, High-resolution TEM (HR-TEM) image (**g**) and the corresponding IFFT image (**h**) taken from the region marked in **f**, showing the blocking effect of the NLRO on dislocation gliding. Bottom-left inset in **g**: the FFT pattern transformed from the corresponding marked region. Top-right inset in **g**: the filtered HR-TEM image from the corresponding marked region. **i**, BF-TEM micrograph showing the high dislocation density between the slip bands. **j**, The enlarged figure from the corresponding red dashed box in **i**. **k**,The evolution of dislocation density as a function of plastic strain, compared with ordering-free CoNiV(SS) and CoNiV-AlTi(SS) samples and the SRO-domain-containing CoNiV(SSA) sample. Scale bars, 200 nm (**a**,**j**,**k**,inset), 5 nm (**b**,**c**,**d**,**g**,**h**), 100 nm (**e**), 50 nm (**f**), 500 nm (**i**).

**Extended Data Fig. 8:**
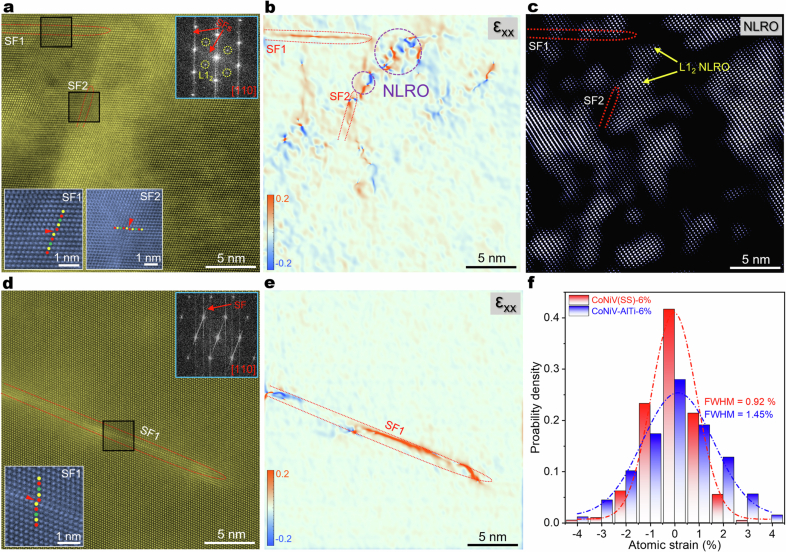
Comparison of atomic strain distribution of the 6% cryogenically strained (a-c) CoNiV-AlTi and (d-e) CoNiV(SS). (a) and (d) The HAADF-STEM images of the selected regions. The top-right insets: the corresponding FFT patterns. The bottem-left inserts: the enlarged regions marked by black boxes, displaying the atomic structure of SFs. (b) and (e) The corresponding atomic elastic strain mapping (horizontal direction (*ε*_xx_) strain along (002) plane). (c) The IFFT image corresponding to (a) obtained from the L1_2_ diffraction spots, showing the distribution of the L1_2_ phase. These are additional results acquired from different samples from those shown in Fig. 3, again demonstrating a high stress concentration close to the intersection point between NLRO and stacking faults in CoNiV-AlTi. (f) The atomic-strain probability density of the two samples. The full width at half maximum (FWHM) of each curve represents the fluctuation degree of the atomic-strain in the corresponding sample.

Next, we unravel the cryogenic strain-hardening mechanisms and the associated effects of NLRO domains in our CoNiV-AlTi alloy. For this purpose, we deform the sample at different strain levels and probe the substructure by TEM. Planar-slip substructures consisting of numerous stacking faults (SFs) along the {111} planes are observed at a small strain of 6% (Fig. 3a). We frequently observe strong L1_2_ diffraction signals at regions in-between two SFs (see a typical micrograph in Fig. 3b). The IFFT analysis (inset in Fig. 3b) transformed from such reflection signals reveals the existence of relatively large, structurally intact (that is, undestroyed by dislocation slip) NLRO domains in these regions. We further analyse the elastic strain fields for the region shown in Fig. 3b, containing two consecutive SFs and a large NLRO domain in-between. Notably, a high elastic strain concentration is observed at the end of the SF, where the SF interacts with the NLRO domain (marked by a dashed red circle in Fig. 3c,d). Similar phenomena have also been observed in other regions of different grains, whereas such elastic strain concentration is not observed in the ordering-free CoNiV(SS) sample deformed to the same strain level (Extended Data Fig. 8). Hence, this feature strongly suggests a blocking effect of NLRO on the motion of partial dislocations.

**Extended Data Fig. 9:**
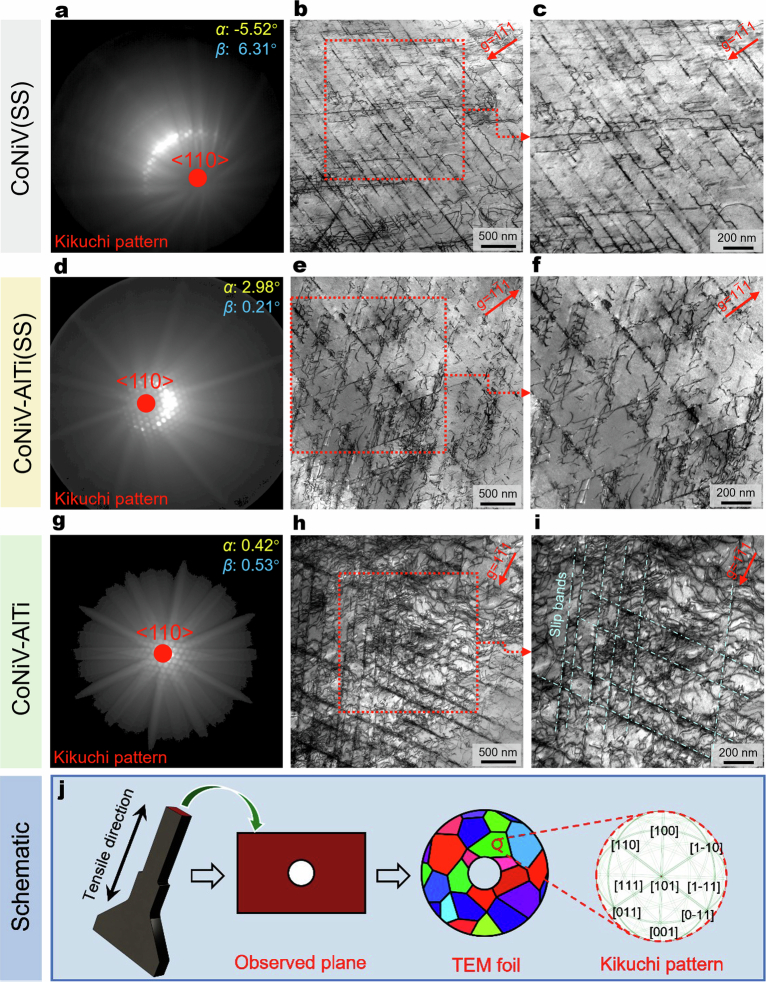
Comparison of dislocation patterning among (a-c) CoNiV(SS), (d-f) CoNiV-AlTi(SS) and (g-i) CoNiV-AlTi samples deformed to 15% strain at 87 K. Samples were probed based on the ${\boldsymbol{g}}=1\bar{1}1$ diffraction vector from grains with a similar orientation relative to the loading direction. Sample preparation procedures are schematically illustrated in (j). For each grain probed, the corresponding Kikuchi pattern was firstly acquired to confirm the orientation of this grain to be close to <110> direction. The presented Kikuchi patterns are taken when keeping TEM stage not tilted. The *α* and *β* values inset in each image represent the TEM stage tilt angles, which indicates the tilt angles we need to take to get the <110> zone axis. In comparison to two solid-solution treated CoNiV(SS) and CoNiV-AlTi(SS) samples, more non-planar dislocations are observed to be originated from well-developed planar-slip bands in the CoNiV-AlTi containing dual-scale ordering at this strain.

**Extended Data Fig. 10:**
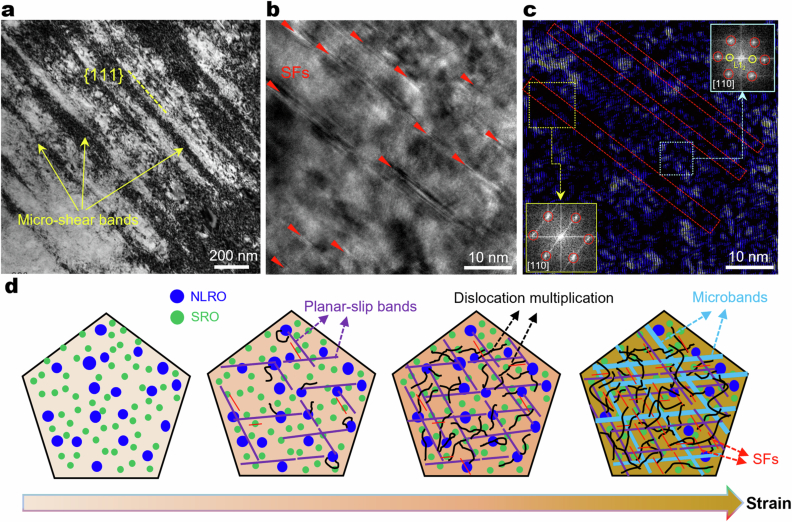
Deformation microstructure of the CoNiV-AlTi alloy cryogenically deformed to 30%. (a) BF-TEM micrograph of the CoNiV-AlTi alloy cryogenically deformed to 30% strain, showing a high density of micro-shear bands. (b) HR-TEM image and (c) the corresponding IFFT image of a local region in (a), showing dense slip bands consisting of stacking faults within micro-shear bands and the destruction of NLRO by the stacking faults, which indicates that the gliding of dislocations through the ordered lattice during straining causes a gradual shear-enhanced order-disorder phase transition. (d) Schematic diagram illustrating the plastic deformation mechanism in the CoNiV-AlTi alloy containing dual-scale chemical ordering.

With further deformation (for example, at 15% strain), a considerable fraction of non-planar dislocation slip is observed between slip bands (Fig. 3e). This is a unique feature that is not observed in the ordering-free CoNiV(SS) sample or the CoNiV-AlTi(SS) sample, when comparing the deformation microstructure of grains with similar orientation relative to the loading direction (Extended Data Fig. 9). These non-planar dislocations are typically emitted at the points where SFs encounter the NLRO domains and experience a high resistance for their further motion (as shown in Fig. 3g,h). The pinning of the leading partials by NLRO can serve as starting points for partial dislocation recombination, double cross-slip and Frank–Read sources, resulting in the nucleation of new dislocation loops, as shown in Fig. 3f. The active gliding of these non-planar dislocations indicates the occurrence of cross-slip of screw dislocations at this strain level (see more detailed dislocation analysis in Supplementary Figs. 7 and 8). This finding is further supported by the presence of a higher fraction of screw dislocations in this alloy compared with the reference ordering-free CoNiV(SS) and CoNiV-AlTi(SS) samples as well as the SRO-domain-containing CoNiV(SSA) sample at the same strain level (revealed by the higher dislocation character parameter *q*: 1.9 for the CoNiV-AlTi sample than the other three samples, determined based on the neutron diffraction data; see Supplementary Figs. 9 and 10). This effect releases local stress concentrations that would otherwise build up owing to deformation localization^28–30^. Further gliding of these dislocations will eventually trigger their mutual interactions and the interaction with the existing slip bands, resulting in a high density of dislocation networks between the dislocation bands (as shown in the microstructure at 30% strain demonstrated in Fig. 3i,j). The influence of NLRO domains on dislocation evolution and patterning is schematically illustrated in Extended Data Fig. 10d.

The deformation-driven evolution of the dislocation density of the investigated materials is shown in Fig. 3k. The NLRO-containing CoNiV-AlTi sample shows a higher rate of dislocation multiplication compared with the reference samples with and without SRO. The more rapid dislocation multiplication, owing to the blocking effect of NLRO on partial dislocation gliding and thus the necessity for new dislocation generation and the subsequent dislocation interactions, serves as an important contribution to the higher strain-hardening rate at cryogenic temperature (Fig. 2a). According to the Taylor hardening law^31,32^, the stress increase owing to strain-hardening can be expressed as: ${\sigma }_{{\rm{f}}{\rm{l}}{\rm{o}}{\rm{w}}}-{\sigma }_{{\rm{Y}}{\rm{S}}}={a}_{0}MGb\sqrt{{\rho }_{\text{d}}}$, where *σ*_flow_ and *σ*_YS_ are the flow stress at a given plastic strain and the yield strength, respectively, *ρ*_d_ is the dislocation density (the initial dislocation density of recrystallized samples can be neglected), and *α*_0 _(dimensionless constant), *G* (shear modulus), *M* (Taylor factor) and *b* (magnitude of the Burgers vector) are regarded as constants that do not vary much during tensile deformation^33,34^. On the basis of the measured dislocation density (Fig. 3k), the deformation-induced stress increase for the NLRO-domain-containing CoNiV-AlTi sample is estimated to be 1.29-times higher than that of the ordering-free CoNiV(SS) sample, at the same global strain of 30%. This value is very close to the result directly acquired from the tensile tests (about 1.26), which confirms the dominant role of NLRO-domain-affected dislocation multiplication on strain hardening of the studied materials.

The traditional view on the promoting role of small-scale local ordering on materials’ strain hardening is based on the shearing of ordered phases by gliding dislocations^13,35,36^. The associated local destruction of the ordered structure produces a glide-plane softening effect driven by the energetically favourable elimination of the introduced high-energy anti-phase boundaries^36^. Such an effect promotes dislocation planar slip by facilitating trailing dislocation gliding on the same slip planes^16,36^. Although we do observe such a dislocation shearing phenomenon and the resulting destruction of NLRO domains along the gliding paths at higher strains (for example, 30%; Extended Data Fig. 10a–c), we believe that this mechanism results in only a moderate influence on the alloy’s strain-hardening rate, owing to the following two factors. First, owing to the reduced SFE at cryogenic temperatures^5,37^, planar dislocation slip is already the prevalent deformation mode for the CoNiV-based alloys, regardless of the existence of local ordering (Extended Data Fig. 9). Second, the existence of SRO domains is expected to have an effect on SFE and dislocation slip planarity^16^, but by itself does not promote a notable increase in the strain-hardening rate of the CoNiV(SSA) sample at cryogenic temperature (Fig. 2a, the comparison between CoNiV(SS) and CoNiV(SSA) samples). In addition, owing to the extremely small size (below 1 nm), SRO can be readily destroyed by only a few pairs of dislocations once they are nucleated and start to glide^18^. Thus, SRO domains are not expected to significantly influence dislocation patterning during plastic deformation in the investigated alloys. This explains the similar evolution of the overall dislocation density observed between the SRO-domain-containing CoNiV(SSA) sample and the ordering-free CoNiV(SS) sample at the same strain level (Fig. 3k). This deficiency of SRO domains as a stand-alone hardening mechanism can be compensated by the additional introduction of NLRO phases. The latter mechanism also allows to alter the dislocation pattern and population, but without producing substantially heterogeneous stress fields.

**Fig. 4:**
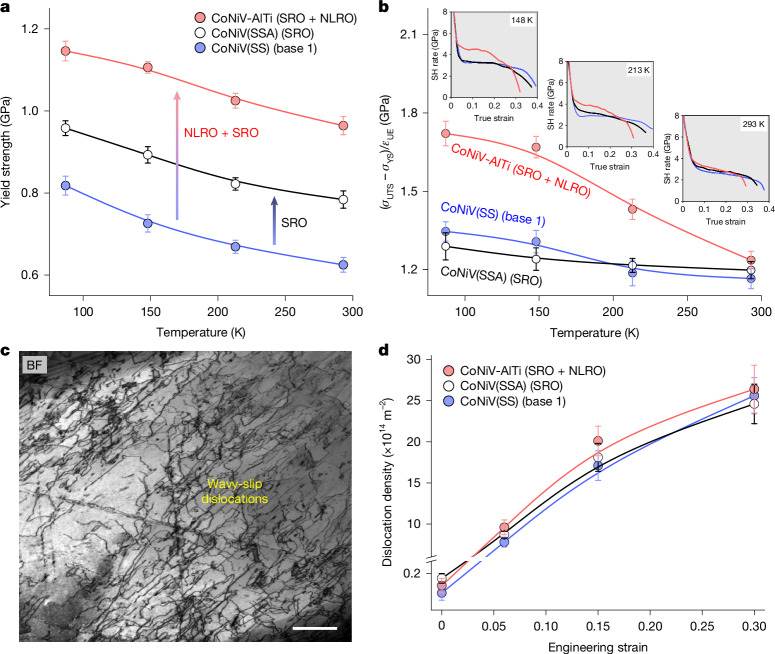
The temperature-dependent mechanical behaviour of samples containing different ordering structures (CoNiV-AlTi, CoNiV(SS) and CoNiV(SSA) samples). **a**, The relation between yield strength and testing temperature. **b**, The value of (*σ*_UTS_ − *σ*_YS_)/*ε*_UE_ (characterizing the alloy’s strain-hardening (SH) ability, where *σ*_UTS_ and *ε*_UE_ are ultimate tensile strength and uniform elongation, respectively) as a function of testing temperature. **c**, BF-TEM image of the NLRO-containing CoNiV-AlTi sample deformed to 6% strain at room temperature. **d**, The deformation-induced evolution of the dislocation density for the three samples strained at room temperature. The error bars represent standard deviation. Scale bar, 500 nm (**c**).

We further evaluate the temperature dependence of the effectiveness of such a microstructural approach. Mechanical tests show that compared with the CoNiV(SS) and CoNiV(SSA) reference samples, the combination of SRO and NLRO domains can elevate the alloys’ yield strength at temperatures up to ambient temperature (Fig. 4a). However, the effects on strain-hardening capability gradually vanish with higher testing temperatures (Fig. 4b), similar to some alloys containing coherent nanoprecipitates^38,39^, indicating a weaker interaction between NLRO domains and dislocations. Unlike the deformation substructure observed at cryogenic temperature, the deformation mode of the CoNiV-AlTi alloy at room temperature shows a limited planar dislocation slip behaviour, and, instead, wavy dislocation slip is prevalent at this temperature owing to the increased SFE (as shown in Fig. 4c). The higher tendency for dislocations to cross-slip at increased temperatures^40,41^, as well as the temperature-dependent anti-phase boundary energy^42^, decreases their interaction with the NLRO obstacles, which means that the presence of NLRO domains has little influence on dislocation multiplication and patterning at elevated temperatures. This is strongly supported by the nearly unchanged evolution of dislocation density between the SRO + NLRO-domain-containing CoNiV-AlTi sample and the ordering-free CoNiV(SS) sample deformed to a same strain level at room temperature (Fig. 4d). This analysis reveals a strong dependence of ordering-induced strain-hardening enhancement on testing temperature, SFE and the associated dislocation gliding modes, which provides a fundamental guideline for the selection of material systems and their operating scenarios that can benefit from our ordering and microstructure design approach.

To conclude, we show here that a dual-scale short- to long-range chemical ordering strategy can substantially improve the mechanical properties of metallic alloys at cryogenic temperatures. The two types of ordered structure confine the dislocations’ mean free path and provide both a higher friction stress (lending strength) and a more rapid dislocation multiplication rate (enhancing the strain-hardening ability) at cryogenic temperatures, as opposed to reference samples without the presence of such an ordering hierarchy. This (sub)nanoscale microstructure strategy is different from the widely adopted precipitation hardening approach, which creates local stress peaks that can lead to damage initiation. We have also validated the effectiveness of the dual-scale chemical ordering approach on improving the cryogenic strength–ductility synergy in NiCrFe-based and CoCrNi-based MEAs (as shown in Supplementary Figs. 11 and 12), demonstrating the generality of our concept. Our work hence widens the microstructure design toolbox that so far lacked effective means for enabling alloys to withstand harsh cryogenic environments.

## Methods

### Materials preparation

The CoNiV and CoNiV-AlTi alloys were cast in a vacuum induction furnace using pure metallic ingredients (purity higher than 99.8 wt%) under a high-purity argon atmosphere. The ingots were then homogenized in vacuum at 1,100 °C for 24 h and cold-rolled into sheets with an 80% reduction in thickness. The solid-solution treatment for the CoNiV-AlTi and the CoNiV alloys was kept at 1,100 °C for 2 min and for 2.5 min followed by water quenching, respectively, to guarantee a similar grain size. The high solid-solution annealing temperature was selected to achieve a (nearly) disordered solid solution in both alloys. The ageing treatment for the CoNiV-AlTi and CoNiV(SSA) samples was carried out at 750 °C for 1 h followed by air cooling to produce the ordering structure. The exact chemical composition of the two alloys, measured by inductively coupled plasma mass spectrometry, is Co_32.5_Ni_33.4_V_34.1_ and Co_31.6_Ni_31.9_V_32.3_Al_2.1_Ti_2.1_ (at%), respectively.

### Microstructural and mechanical characterization

Electron backscatter diffraction measurements were performed using a Zeiss-Cross Beam XB 1540 focused ion beam-scanning electron microscopy instrument. Aberration-corrected TEM characterization was performed using a ThermoFisher Themis Z equipped with 2 aberration correctors under 300 kV. Atomic-strain maps were obtained by using the geometrical phase analysis method^43^. For each sample, at least five atomic-resolution HAADF-STEM images were collected to validate the reproducibility of the atomic-strain results. The sample for the statistics of number density was prepared using a typical focused ion beam lift-out method using Zeiss-Cross Beam 350 dual-beam system, and the thickness of the interested area was estimated to about 100 nm. The size distribution of the SRO, NLRO and L1_2_ precipitates was analysed using Image J software. TEM specimens for the study of deformation microstructure were prepared using electro-polishing with a solution of 20 vol% perchloric acid and 80 vol% acetic acid, at 258 K and 50 mA. Dislocation patterns were characterized using a ThermoFisher Talos F200X operated at an accelerating voltage of 200 kV. The determination of SFE was based on the spacing between two dissociated Shockley partial dislocations using the DF-STEM images under the condition of weak-beam diffraction (more details in Supplementary Note 2 and Supplementary Fig. 13).

APT experiments were conducted primarily to study the composition and structure of the ordering phases^44^. The orientation-specific preparation procedure used for APT needle-like specimens was performed using an FEI Helios Nanolab 660 dual-beam system. The analysis of the APT data was performed using IVAS 3.8.4 software. Typical time-of-flight mass spectra with identified ions for the investigated alloy systems are provided in Supplementary Fig. 14. APT experiments were operated in voltage pulsing mode with a pulse rate of 200 kHz, a pulse fraction of 20%, a specimen temperature of 70 K and a detection rate of 5 ions per 1,000 pulses. An ML-APT analysis method was used to identify the existence and crystal structure of SRO domains in our investigated materials, using at least two APT tips for each grain orientation. More details of the ML-APT methodology can be found in Supplementary Note 3 and refs. ^45,46^.

Tensile specimens, with a gauge length of 8.5 mm, a width of 1.8 mm and 1.2 mm in thickness, were machined along the rolling direction from the cold-rolled and heat-treated sheets. At least five tensile specimens were tested to ensure the repeatability. Quasi-static uniaxial tensile tests were performed with an adjustable environment chamber at different temperatures (293 K, 213 K, 148 K and 87 K) at a nominal engineering strain rate of 1 × 10^−3^ s^−1^, and the strain was measured by a mechanical extensometer (epsilon LHT). Compact-tension specimens for the fracture toughness tests were prepared in accordance with ASTM E1820^47^. They had dimensions of width *W* = 20 mm and thickness *B* = 5.5 mm and a notch length of about 8.5 mm. The fatigue pre-cracks were created under load control at a stress intensity range (Δ*K*) of about 15 MPa m^0.5^ and a load ratio (*R*) of 0.1 at a frequency of 10 Hz on an Instron 8801 universal loading machine. The final pre-crack length (*a*_p_) was no less than 10 mm in length with an approximate *a*_p_/*W* of 0.5 to ensure a pre-crack length well above the minimum length (about 1.3 mm) required by ASTM E1820^47^. More details of fracture toughness testing and the associated results can be found in Supplementary Note 4. At least two fracture toughness specimens for each sample were tested to ensure the repeatability.

### Neutron diffraction characterization

Neutron diffraction tests were carried out using a general-purpose powder diffractometer (https://csns.cn/31113.02.CSNS.GPPD) at the China Spallation Neutron Source (https://csns.cn/31113.02.CSNS). The power of the neutron source was 120 kW. All undeformed samples for neutron diffraction measurement were mechanically polished. For deformed samples, the gauge length of the interrupted tensile samples was used to evaluate the dislocation density of different tensile strains. Data were collected using the time-of-flight method with each sample measured for 1 h. The samples were rotated with an axis perpendicular to the incident neutron beam to eliminate the influence of textures. Quantitative analysis on the fraction of phases and lattice parameters (*a*) was conducted based on Rietveld method using the GSAS-II program. The background was taken as the base to better display the peaks or refinement results in all neutron diffraction patterns. Lattice misfit (*δ*) between the matrix and LRO domain/phases was calculated using the following relation^6^: *δ* = 2 × (*a*_LRO_ − *a*_Matrix_)/(*a*_LRO_ − *a*_Matrix_). The dislocation density of the presented alloys was calculated by the modified Williamson–Hall and modified Warren–Averbach methods^48^ based on the neutron diffraction profiles, and details for the calculation are described in Supplementary Note 5.

### Thermodynamic calculations

The equilibrium phase fractions as a function of temperature were calculated using the Thermo-Calc software together with the TCHEA4 thermodynamic database for HEAs using the global minimization of the Gibbs energy of the system. The enthalpy of mixing of different elements in the fcc phase with CoNiV composition at 1,100 °C and the pseudo-ternary phase diagram at 750 °C was calculated assuming that *N*(Ni) = *N*(Co) = *N*(V), therefore the tie-lines are out of the plane of the diagram and the phase boundary should be interpreted only as a borderline composition for the L1_2_ formation.

## Online content

Any methods, additional references, Nature Portfolio reporting summaries, source data, extended data, supplementary information, acknowledgements, peer review information; details of author contributions and competing interests; and statements of data and code availability are available at 10.1038/s41586-025-09458-1.

## Supplementary information

Supplementary Information This Supplementary Information file contains Supplementary Notes 1–5, Figs. 1–16, Tables 1–5 and References.

## Data Availability

The data that support the findings of this study are available from the corresponding authors upon reasonable request.
