# Supplementary material for: Dual-scale chemical ordering for cryogenic properties in CoNiV-based alloys
Source: Nature. 2025 Aug 27;645(8080):385–91. doi: 10.1038/s41586-025-09458-1 (PMC12422977; doi:10.1038/s41586-025-09458-1)
Supplement: Supplementary file 1 — This Supplementary Information file contains Supplementary Notes 1–5, Figs. 1–16, Tables 1–5 and References. [file 41586_2025_9458_MOESM1_ESM.pdf]

nature portfolio

https://doi.org/10.1038/s41586-025-09458-1

Supplementary information

Dual-scale chemical ordering for cryogenic properties in CoNiV-based alloys

In the format provided by the authors and unedited

Supplementary Material:

Dual-scale chemical ordering for cryogenic properties in CoNiV-based alloys

Tiwen Lu[a†], Binhan Sun[a†], Yue Li[b], Sheng Dai[c], Ning Yao[a], Wenbo Li[c], Xizhen Dong[b], Xiyu Chen[a], Jiacheng Niu[d], Fan Ye[c], Alisson Kwiatkowski da Silva[b], Shuya Zhu[e], Yu Xie[a], Xiaofeng Yang[a], Sihao Deng[f], Jianping Tan[a], Zhiming Li[e], Dirk Ponge[b], Lunhua He[f,g], Xian-Cheng Zhang[a*], Dierk Raabe[b*], Shan-Tung Tu[a]

[a]Key Laboratory of Pressure Systems and Safety, Ministry of Education, East China University of Science and Technology, Shanghai 200237, China

[b]Max-Planck-Institut für Sustainable Materials, Max-Planck-Straße 1, 40237 Düsseldorf, Germany

[c]Key Laboratory for Advanced Materials and Joint International Research Laboratory of Precision Chemistry and Molecular Engineering, Feringa Nobel Prize Scientist Joint Research Centre, School of Chemistry and Molecular Engineering, East China University of Science & Technology, Shanghai 200237, China

[d]National Engineering Research Center of Near-net-shape Forming for Metallic Materials, South China University of Technology, Guangzhou, Guangdong 510640, China

[e]School of Materials Science and Engineering, Central South University, Changsha 410083, China

[f]Spallation Neutron Source Science Center, Dongguan 523803, China

[g]Beijing National Laboratory for Condensed Matter Physics, Institute of Physics, Chinese Academy of Sciences, Beijing 100190, China

[†]These authors contributed equally to this work.

*Corresponding author. E-mail: xczhang@ecust.edu.cn (X.-C. Z.); d.raabe@mpie.de (D. R.)

Contents

Supplementary Note 1 | The contribution of chemical local ordering on the strength of the CoNiV-AlTi alloy 3
Supplementary Note 2 | The measurement of stacking fault energy 5
Supplementary Note 3 | Machine learning enhanced atom probe tomography analysis 7
Supplementary Note 4 | The testing method and calculation procedure of fracture toughness........ 8
Supplementary Note 5 | Calculation of dislocation density 10
Supplementary Fig. 1 | The cryogenic tensile properties of the investigated alloys treated at different temperatures from 500 to 850 ˚C and corresponding evolution of ordering 12
Supplementary Fig. 2 | Secondary electron (SE) image and energy-dispersive X-ray (EDX) spectroscopy result of the CoNiV-AlTi sample 13
Supplementary Fig. 3 | Calculated formation energy of different ordering structures of the CoNiV material at 750 °C. 14
Supplementary Fig. 4 | The microstructure and fracture morphology of the CoNiV-AlTi(24h aged) sample. 15
Supplementary Fig. 5 | EBSD-inverse pole figures (IPFs) of (a) the CoNiV(SS) and (b) the CoNiV(SSA) samples. 16
Supplementary Fig. 6 | Microstructure and cryogenic tensile property of CoNiV-AlTi sample undergoing different solid solution time at 1100 °C 17
Supplementary Fig. 7 | The deformation microstructure of CoNiV-AlTi sample at the same diffraction vector under the two different zone axes 18
Supplementary Fig. 8 | The dislocation distribution in the CoNiV-AlTi sample 19
Supplementary Fig. 9 | Qualitative assessment of dislocation types for four samples. 20
Supplementary Fig. 10 | Neutron diffraction patterns and the calculation of dislocation density. 21
Supplementary Fig. 11 | Cryogenic (87 K) mechanical properties and representative microstructure of the CoCrNi-based alloys with different ordering states. 22
Supplementary Fig. 12 | Cryogenic (87 K) mechanical properties and representative microstructure of the NiCrFe-based alloys with different ordering states 23
Supplementary Fig. 13 | Detailed g·b analysis of partial dislocations in the cryogenically (87 K) strained (6%) samples and the calculation of SFE. 24
Supplementary Fig. 14 | Typical APT time-of-flight mass spectrum with identified ions. (a) the CoNiV-AlTi sample and (b) the CoNiV(SSA) sample. 25
Supplementary Fig. 15 | Schematic diagram of ordered domains sheared by a pair of dislocations.. .. 26
Supplementary Fig. 16 | Typical atomic-scale resolution HAADF-STEM images of the CoNiV(SS) sample. 27
Supplementary Table 1 | Thermal and physical parameters of elements. 28
Supplementary Table 2 | SFE, elastic and mechanical properties of investigated samples 29
Supplementary Table 3 | Details of the data presented in Fig. 2b 30
Supplementary Table 4 | The Burgers vector of dislocations using the *g·b* criterion 32
Supplementary Table 5 | Details of the data presented in Extended Data Fig. 3b 33

Supplementary Note 1 | The contribution of chemical local ordering on the strength of the CoNiV-AlTi alloy

The enhancing effect of chemical local ordering (long-range order and short-range order) on the strength of a compositional complex material mainly lies in its order strengthening effect[1,2]. This is associated with the dislocation shearing of ordering clusters which produces an antiphase boundary that has a high energy opposing the motion of the penetrating dislocations[2,3]. The relation between critical resolved shear stress (CRSS, τ_c) for dislocation gliding and the characteristics of chemical local ordering can be expressed as[4]:

$$2\tau_c b = \gamma_{APB}\left(\frac{d_I}{L_I} - \frac{d_{II}}{L_{II}}\right) \tag{1-1}$$

where γ_{APB} is the antiphase boundary energy of the chemical local ordering domain (for LRO, the value is around the order of 10^{-1} J/m^2 [1,2,5]), d_i and L_i are the average length of one of the paired dislocation lying in ordering particles and the average spacing of the ordering particles along the dislocation, respectively, as schematically shown in the figure below, I denotes the first shearing dislocation which produces an antiphase boundary and II represents the second trailing dislocation that restores the ordering particle. The value of L also equals the mean free path of the dislocation. Supplementary Fig. 15 shows schematic diagram of ordered domains sheared by a pair of dislocations.

This relation reveals that the strengthening effect caused by chemical local ordering is strongly influenced by both the γ_{APB} and L_I; the former parameter describes the resistance of the ordering particle to dislocation shearing (i.e., how easy they can be cut by a dislocation), and the latter parameter highlights the density and dispersion of the ordering particles (i.e., how dense they are distributed). A higher number density and a more densely distributed ordering clusters reduce dislocation mean free path, which can be calculated by[6]

$$L = \bar{d}\left(\sqrt{\frac{\pi}{4f}} - 1\right) \tag{1-2}$$

$$\bar{d} = \sqrt{2/3}\,d \tag{1-3}$$

$\bar{d}$ is the mean radius of a circular cross-section in a random plane crossing a spherical precipitate, d is average diameter of the ordered domains, f is the volume fraction of the ordered domains. The dislocation mean free path produced by short-range ordering (SRO) and nanoscale long-range ordering (NLRO) was calculated to be 1.9 nm and 1.8 nm, respectively.

We also use a widely adopted particle-shearing model to quantify the contribution of NLRO to the yield strength[7,8], which is detailed as follows: The overall strengthening effect associated

with coherent precipitates is determined by the highest value among the following two contributions: the combined strengthening effects from coherency and shear modulus mismatch ($\sigma_{CS} + \sigma_{MS}$) or the atomic ordering strengthening (σ_{OS}). The increase in strength due to the lattice mismatch and shear modulus mismatch between matrix and $L1_2$ precipitates can be estimated by[7]:

$$\Delta\sigma_{CS}+\Delta\sigma_{MS} = M\cdot\alpha_{\varepsilon}\cdot(G\cdot\varepsilon)^{\frac{3}{2}}\cdot\left(\frac{\bar{r}f}{0.5Gb}\right)^{\frac{1}{2}} + M\cdot 0.0055\cdot(\Delta G)^{\frac{3}{2}}\cdot\left(\frac{2f}{G}\right)^{\frac{1}{2}}\cdot\left(\frac{\bar{r}}{b}\right)^{\frac{3m}{2}-1} \qquad (1\text{-}4)$$

where M is the Taylor factor (3.06[10]), α_{ε} is a constant (2.6[10]), G is the shear modulus (e.g., 72.6 GPa for the CoNiV-AlTi), ε is the constrained lattice mismatch (2/3 of the lattice mismatch, δ, determined by neutron diffraction), $\bar{r} = \sqrt{2/3}\cdot r$ is the mean radius size of the precipitate, $b = \frac{\sqrt{2}}{2}a$ is the magnitude of the Burgers vector of the matrix (where a is lattice parameter), ΔG is the shear modulus mismatch between the matrix and $L1_2$ precipitates (~10 GPa[9]), m is a constant taken to be 0.85[7]. The strengthening effect due to the atomic ordering can be approximated by[7,8]:

$$\Delta\sigma_{OS} = M\cdot 0.81\cdot\frac{\gamma_{APB}}{2b}\cdot\left(\frac{3\pi f}{8}\right)^{\frac{1}{2}} \qquad (1\text{-}5)$$

where γ_{APB} is strongly related to its chemical composition (here taken as 0.12 J/m^2, adopted from the Ni_3Al phase in Ni-based superalloys[1,10]). Through the comparison between the two types of strengthening contributions (47 MPa and 234 MPa for $\Delta\sigma_{CS}+\Delta\sigma_{MS}$ and $\Delta\sigma_{OS}$, respectively), we find that the NLRO-induced strengthening is dominated by atomic ordering strengthening (234 MPa). The calculated strengthening from NLRO is slightly higher than that estimated from the experiment (~167 MPa). This discrepancy can be due to the overestimation of γ_{APB} of the NLRO phase in our material, whose composition may be deviating from its stoichiometric thermodynamic composition due to the incomplete element partitioning in the early stage of precipitation[10].

Supplementary Note 2 | The measurement of stacking fault energy

The determination of stacking fault energy (SFE) is based on the spacing between two dissociated Shockley partial dislocations, using the following equation[11,12]:

$$\mathrm{SFE} = \frac{G b_\mathrm{p}^2}{8\pi d_0}\left(\frac{2\text{-}\nu}{1\text{-}\nu}\right)\left(1\text{-}\frac{2\nu\cos(2\beta)}{2\text{-}\nu}\right) \tag{2-1}$$

where ν is the Poisson's ratio (e.g., 0.31 and 0.3 for the CoNiV(SS) and CoNiV-AlTi(SS) samples, respectively). Elastic parameters were measured by means of ultrasonic techniques (Olympus 5900 PR, USA) at room temperature. Two ultrasonic transducers (20 and 100 MHz) were successively placed for measuring the dilatational and shear wave speeds of samples with a thickness of 0.5 mm. We assumed a minimal change in the shear modulus at the cryogenic temperature as seen in similar alloys[13-15]. d_0 is the spacing between two dissociated Shockley partial dislocations, b_p is the magnitude of the Burgers vectors of the partial dislocations (e.g., 0.1471 and 0.1474 nm for the CoNiV(SS) and CoNiV-AlTi(SS) samples, respectively, based on the lattice parameters derived from neutron diffraction data), and β is the angle between the dislocation line and the Burgers vector of the full dislocation.

Two methods were deployed here to determine the separation distance between two partial dislocations; one is based on the dark-field scanning transmission electron microscopy (DF-STEM) images under the condition of weak-beam diffraction[16,17], and the other is based on high-resolution high-angle annular dark field scanning transmission electron microscopy (HAADF-STEM) images[11,18,19]. For the first method, the Burgers vector of the dislocations was determined through the $\boldsymbol{g}\cdot\boldsymbol{b} = 0$ analysis using three independent <220> diffraction vectors (i.e., $\boldsymbol{g}$ = 022, $\boldsymbol{g}$ = 220, and $\boldsymbol{g}$ = 202), from which the Shockley partial dislocations can be identified[12,17,20,21]. For the second method, high-resolution HAADF-STEM images were taken along [110] zone axis to discern the origin of the atomic misfit along the (111) lattice planes, in which the partial dislocations reside, typical HAADF-STEM images shown in Supplementary Fig. 16. More than 20 pairs of partial dislocations were measured and quantified for both methods, and the spacing between two dissociated partial dislocations was measured using the Digital Micrograph software[22]. Specimens that are cryogenically strained to 6% were used for this crystallographic analysis. Representative high-resolution HAADF-STEM and DF-STEM images showing the dissociation of full dislocations in this sample are displayed in figure below and Supplementary Fig. 13, respectively. The SFE of the CoNiV(SS) sample is calculated to be 52.3 ± 6.5 mJ/m^2 (based on DF-STEM images) and 55.5 ± 32.7 mJ/m^2 (based on high-resolution HAADF-STEM images). Apparently, the two methods yield a similar average SFE value but the latter method

which works by using high-resolution HAADF-STEM images has a larger error bar, which is also reported in the literature[19]. Therefore, we select the first method, i.e., measuring the separation distance from the DF-STEM images, to compare the SFE among the CoNiV(SS), CoNiV-AlTi(SS), CoNiV(SSA), and CoNiV-AlTi samples.

Supplementary Note 3 | Machine learning enhanced atom probe tomography analysis

In this work, we employed a machine learning enhanced APT (ML-APT) analysis to investigate the ordering states in SRO-containing CoNiV(SSA) sample and SRO + NLRO-containing CoVNi-AlTi sample.

The detailed information on the developed ML-APT method has been published in our recent work[23,24], which has been successfully applied to the configuration analysis of SRO in Fe-Al and CoCrNi alloys. The basic workflow for the analysis of SRO configurations in CoNiV(SSA) and CoVNi-AlTi samples is as follows: First, we performed correlative scanning electron microscopy (SEM)-electron backscattered diffraction (EBSD)-focused ion beam (FIB)-APT to characterize their microstructure in selected grains along the <111> and <002> orientations. At least two APT tips were prepared along each orientation. As shown in Extended date Fig. 2, the collected APT data along specific orientations with the optimal depth resolution were voxelized into millions of 1-nm cubes that are transformed into spatial distribution maps (z-SDMs) along the depth. Second, an SRO recognition model was obtained utilizing the simulated SRO pattern bank to train 1D convolutional neural networks. Its reliability has been verified by large-scale APT simulations[23,24]. Third, the pre-processed experimental z-SDMs were fed into the SRO recognition model to obtain the 3D SRO distribution. The Pearson contingency coefficient (μ) was used to test the statistical significance of the difference between these distributions of Co-Co, V-V and Ni-Ni pairs and a chemically-randomized dataset. The values of 0 and 1 correspond to the minimum and maximum differences between the two distributions, respectively. We defined a threshold value to classify (non-)randomness at 0.25 for the CoNiV(SSA) sample. Note that different from the equiatomic ternary system, it is necessary to define separate threshold values for each elemental pair in non-equiatomic systems due to the significantly different size distributions observed in the randomized datasets. To figure out the impact of the randomly-formed SRO from the truly random solid solution, we randomly swapped the elemental identities of data points while retaining the original x, y, and z coordinates. Then, the same ML-APT recognition model was applied to the random dataset to obtain the randomly-formed SRO domains.

Supplementary Note 4 | The testing method and calculation procedure of fracture toughness

We evaluated the fracture resistance by measuring the crack-resistance curves (R-curves) with the nonlinear elastic energy release rate[25], the J-integral, as a function of the crack extension, Δa. Tests at 87 K were subjected in displacement control under a constant displacement rate of 0.8 mm/min. A clip gauge of 5 mm (-1/+10 mm) gauge length (Epsilon Technology, Jackson, WY, USA) was used to measure the load-line displacement. Crack lengths, a_i, were calculated from equations provided by ASTM for C(T) samples where the compliance is measured on the load-line:

$$\frac{a_i}{W} = 1.000196 - 4.06319\mu + 11.242\mu^2 - 106.043\mu^3 + 464.335\mu^4 - 650.677\mu^5 \quad (4\text{-}1)$$

$$u = \frac{1}{[B_e E C_{c(i)}]^{0.5} + 1} \quad (4\text{-}2)$$

In Eq. (4-2), B_e is the effective thickness ($B_e = B\text{-}(B\text{-}B_N)^2/B$), B_N is the thickness of the sample at the side groove, E is the material's elastic modulus, and $C_{c(i)}$ is the rotation-corrected, elastic-unloading compliance. Next, for each crack length data point, a_i, the corresponding J_i-integral was calculated as the aggregate of elastic, $J_{el(i)}$, and plastic components, $J_{pl(i)}$, strain energy contributions:

$$J_i = \frac{K_i^2}{E'} + J_{pl(i)} \quad (4\text{-}3)$$

where $E' = E/(1\text{-}v^2)$, v is Poisson's ratio. K_i, the linear-elastic stress intensity corresponding to each data point on the load-displacement curve, was calculated as follows:

$$K_i = \frac{P_i}{(BB_N W)^{0.5}} f(a_i/W) \quad (4\text{-}4)$$

In Eq. (4-4), P_i is the applied load at each individual data point and $f(a_i/W)$ is a geometry-dependent function as listed in the ASTM standard. The plastic component, $J_{pl(i)}$, can be calculated from the following equation:

$$J_{pl(i)} = \left[J_{pl(i-1)} + \left(\frac{\eta_{pl(i-1)}}{b_{(i-1)}}\right)\frac{A_{pl(i)}\text{-}A_{pl(i-1)}}{B_N}\right]\left[1\text{-}\gamma_{(i-1)}\left(\frac{a_{(i)}\text{-}a_{(i-1)}}{b_{(i-1)}}\right)\right] \quad (4\text{-}5)$$

where $\eta_{pl(i-1)} = 2 + 0.522\, b_{(i-1)}/W$ and $\gamma_{pl(i-1)} = 1 + 0.76\, b_{(i-1)}/W$. $A_{pl(i)} - A_{pl(i-1)}$ is the increment of plastic area under load-displacement curve. b_i is the uncracked ligament width (i.e., $b_i = W - a_i$). J_i can be determined from the corresponding crack extension ($\Delta a = a_i - a$) using the above equation.

All the samples in the present study satisfy the J dominance condition in plane strain, i.e., the uncracked ligament b and thickness $B > 10(J_Q/\sigma_0)$, where J_Q is the conditional toughness calculated by the intersection of the blunting line with the R-curve, and σ_0 is the flow stress (the

average of the yield and ultimate tensile stress). The fracture toughness, K_{JIc}, expressed in terms of the stress intensity then can be calculated using the standard *J*-*K* equivalence (mode I) correlation $K_{JIc} = (E'J_{Ic})^{1/2}$. The test results are shown in Extended data Fig. 3.

Supplementary Note 5 | Calculation of dislocation density

The evolution of dislocation density in the presented alloys at different strains was calculated by the combination of modified Williamson-Hall (MWH) and modified Warren-Averbach (MWA) methods[26] based on the neutron diffraction data. According to the MWH method, the full width at half maximum (FWHM), ΔK, of the diffraction peaks is a function of the average crystallite size (D) peaks and the dislocation density (ρ) as follows[27]:

$$\Delta K = \frac{0.9}{D} + \left(\frac{\pi A^2 b^2}{2}\right)^{\frac{1}{2}} \rho^{\frac{1}{2}} \left(K\overline{C}^{\frac{1}{2}}\right) + \mathrm{O}(K^2\overline{C}) \tag{5-1}$$

where K represents the magnitude of Bragg position, directly obtained from the profiles, A is a constant depending on the effective outer cutoff radius of dislocations, O represents the high order terms which can be neglected for calculation. Based on the neutron diffraction peak information, the K and ΔK ($\Delta K_{\mathrm{measure}}$) of each diffraction peak can be calculated. It is worth noting that the actual FWHM of the sample ($\Delta K_{\mathrm{sample}}$) is not equal to $\Delta K_{\mathrm{measure}}$, and the peak width ($\Delta K_{\mathrm{instru}}$) caused by the instrument itself needs to be removed. The $\Delta K_{\mathrm{instru}}$ was calibrated with a standard Si sample.

$$\overline{C} = \overline{C}_{\mathrm{h00}}\left(1 - qH^2\right) \tag{5-2}$$

$$H^2 = \frac{h^2k^2 + k^2l^2 + h^2l^2}{\left(h^2 + k^2 + l^2\right)^2} \tag{5-3}$$

$$\overline{C}_{\mathrm{h00}} = a_1[\,1 - \exp(-\frac{A_{\mathrm{i}}}{b_1})] + c_1A_{\mathrm{i}} + d_1 \tag{5-4}$$

$$q = a_2[\,1 - \exp(-\frac{A_{\mathrm{i}}}{b_2})] + c_2A_{\mathrm{i}} + d_2 \tag{5-5}$$

$$A_{\mathrm{i}} = 2C_{44}/(C_{11} - C_{12}) \tag{5-6}$$

$$\frac{[(\Delta K)\text{-}(0.9/D)]^2}{K^2} = \left(\frac{\pi A^2 b^2}{2}\right) \overline{C}_{\mathrm{h00}}\left(1 - qH^2\right) \tag{5-7}$$

where $\overline{C}_{\mathrm{h00}}$, q, a_{i}, b_{i}, c_{i} and d_{i} are constants related to the anisotropic elastic constants (C_{11}, C_{12} and C_{44}) and dislocation characters[28]. An extensive literature research on the elastic constants of face-centered cubic (FCC) CoNi-based medium-entropy alloys shows that there is no significant difference in A_{i} among these alloys[29-32]. Therefore, C_{11}, C_{12} and C_{44} (296 GPa, 202 GPa and 176 GPa, respectively) taken from a CoCrNi MEA[30] were used. q is a variable related to the fraction of screw and edge dislocations[33], which can be determined experimentally by the linear regression of the $[(\Delta K) -(0.9/D)]^2/K^2$ versus H^2, as shown in Eq. (5-7), *hkl* represents the crystal indices of FCC system. According to the anisotropic elastic constants and the determination of a_2, b_2, c_2 and d_2[28], the theoretical value of q is ~1.65 and ~2.45 for edge dislocations and screw

dislocations in CoNi-based medium-entropy alloys (also reflected in Supplementary Fig. 9), respectively. q value can be regarded to be independent with the strain during deformation[34].

Notably, A, dislocations distribution parameter, is dependent on the effective outer cutoff radius of dislocations (R_e). There is no direct experimental method available to determine A. It can only be obtained on the basis of the relation $A = R_e\rho^{1/2}$, where R_e can be determined by applying the MWA method. According to the MWA method, R_e and ρ can be estimated by Fourier analysis as:

$$\ln A(L) = \ln A^s(L) + Y(L)\,(K^2\overline{C}) + O\,(K^2\overline{C}) \tag{5-8}$$

$$A(L) = \exp(-(0.5L^2(W^G)^2 + LW^L)) \tag{5-9}$$

$$Y(L) = -(\frac{\rho\pi b^2 L^2}{2})\,\ln(\frac{R_e}{L}) \tag{5-10}$$

where L, $A(L)$, $A^s(L)$ represent the Fourier length, the real part of the Fourier coefficient and size Fourier coefficient, respectively, W^L and W^G represent FWHM of the Lorentzian function and the Gaussian function[35,36], respectively. Further, Eq. (5-10) can be rewritten as:

$$\frac{Y(L)}{L^2} = \left(\frac{\rho\pi b^2}{2}\right)\ln L - (\frac{\rho\pi b^2}{2})\ln(R_e) \tag{5-11}$$

where the values of $Y(L)$ for different L were acquired through the procedure of fitting $\ln A(L)$ against $K^2\overline{C}$. The determination of $A(R_e\rho^{1/2})$ can be achieved by means of linear regression analysis on the linear section of $\frac{Y(L)}{L^2}$ and $\ln L$ data, and a typical fitting process can be seen. After the calculation for each strain, the value of A is within the range of 0.6-0.8 at 87 K and 298 K (see a typical example shown in Supplementary Figs. 10 c and d). This is consistent with some previous literature which reported that A underwent no significant change during tensile deformation[34,37-39]. Finally, the best linear fitting between ΔK and $K\overline{C}^{1/2}$ for each strain can be used to determine the value of ρ.

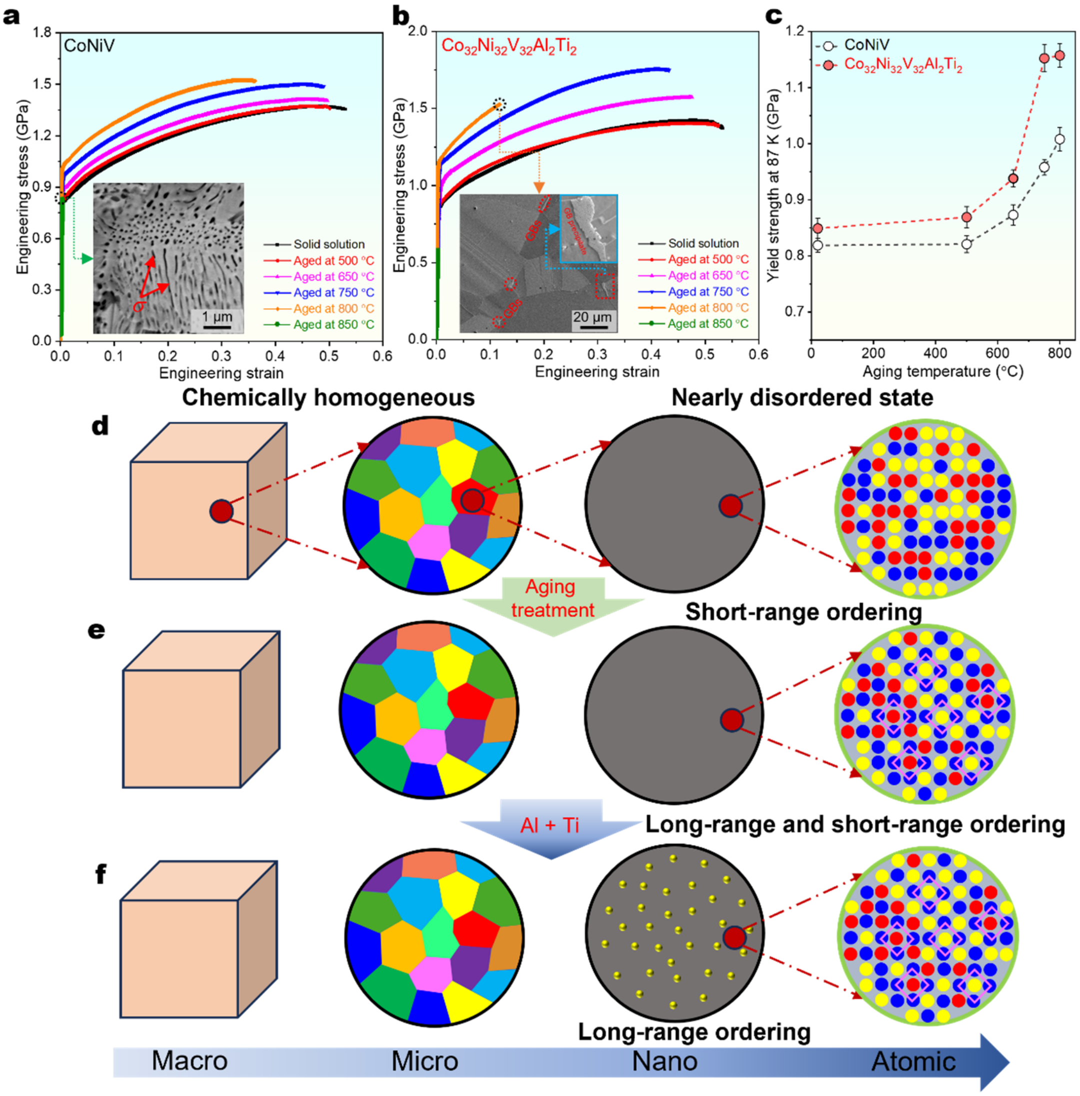

Supplementary Fig. 1 | The cryogenic tensile properties of the investigated alloys treated at different temperatures from 500 to 850 ˚C and corresponding evolution of ordering. (a) CoNiV. The inset is the microstructure after aging at 850 ˚C, showing the formation of the brittle σ phase; (b) $Co_{32}Ni_{32}V_{32}Al_2Ti_2$ (at.%). The inset is the microstructure after aging at 800 ˚C, where the formation of large secondary phases at grain boundaries (GBs) are seen. (c) Yield strength of the two samples as a function of the aging temperature. Schematic diagram of the relationship among chemical composition, heat treatment conditions and microstructure from macro to atomic scales: (d) CoNiV(SS), (e) CoNiV(SSA), (f) CoNiV-AlTi.

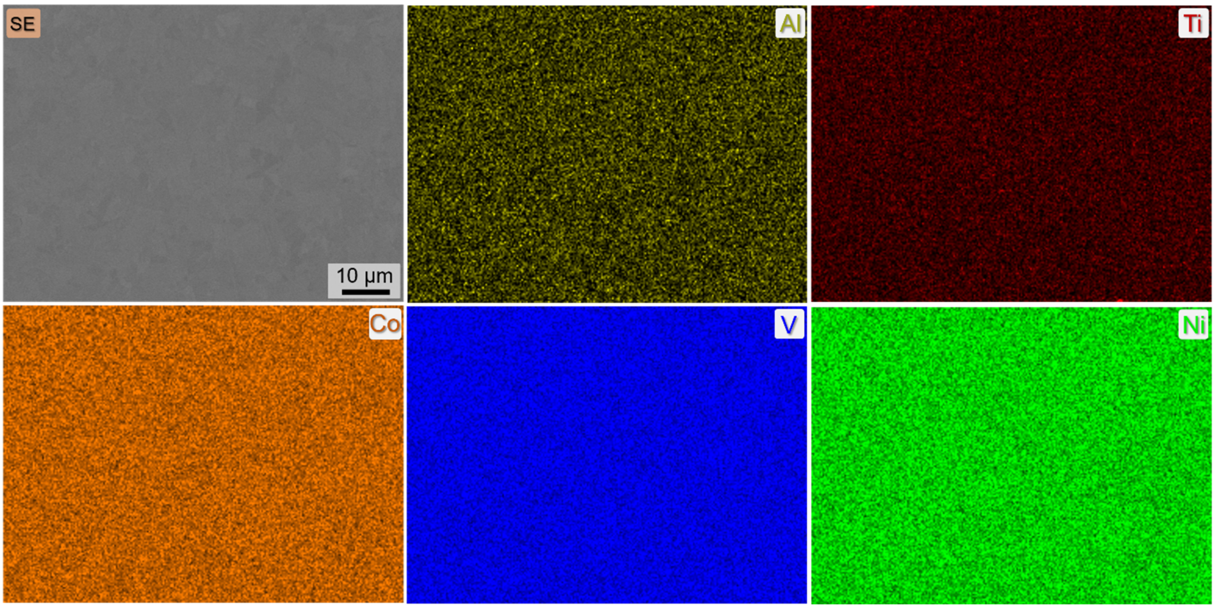

Supplementary Fig. 2 | Secondary electron (SE) image and energy-dispersive X-ray (EDX) spectroscopy result of the CoNiV-AlTi sample, indicating that the chemical composition is homogeneous after heat treatment.

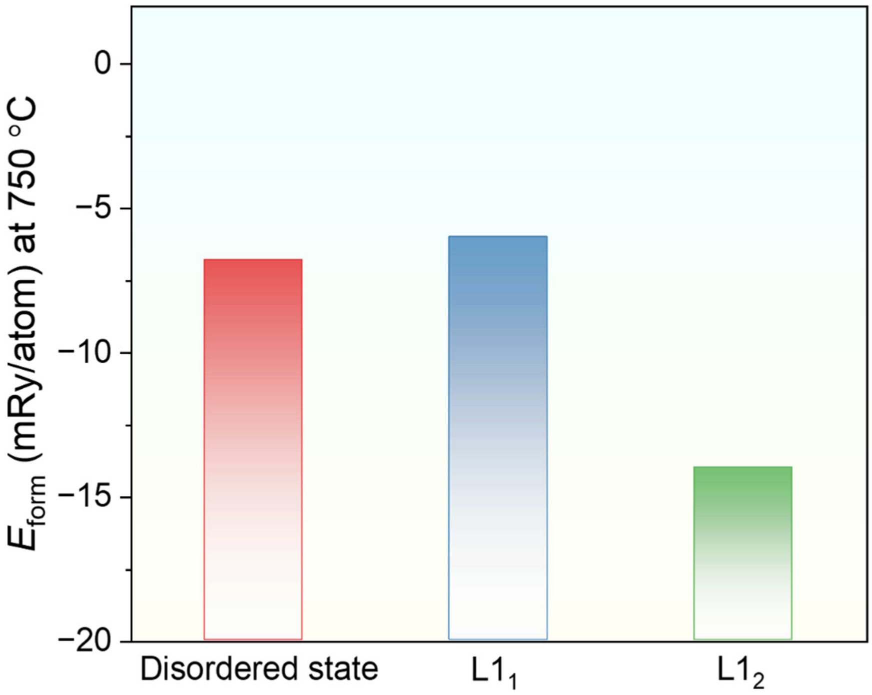

Supplementary Fig. 3 | Calculated formation energy of different ordering structures of the CoNiV material at 750 °C. The calculation was based on the density functional theory (DFT) calculation[40]. E_{form} represents the formation energy of disordered state, $L1_1$ and $L1_2$. Based on the literature[40], the formation energy E_{form} of CoNiV MEA can be calculated by $E_{form}=G^{\alpha}_{MEA}-\frac{1}{3}G^{bcc}_{V}-\frac{1}{3}G^{hcp}_{Co}-\frac{1}{3}G^{fcc}_{Ni}$, where G^{α}_{MEA} is the Gibbs free energy of CoNiV MEA with α (disordered, $L1_1$, $L1_2$) structure. G^{bcc}_{V} is the Gibbs free energy of V with BCC structure, G^{hcp}_{Co} is the Gibbs free energy of Co with hexagonal close-packed (HCP) structure, G^{fcc}_{Ni} is the Gibbs free energy of Ni with FCC structure. These values were acquired based on DFT calculations[40]. $L1_2$ exhibits the lowest formation energy at the temperature of 750 °C, and the E_{form} of $L1_1$ and disordered state is similar. Although the E_{form} of $L1_1$ is slightly higher than that of disordered state at 750 °C, the formation of $L1_1$ within CoNiV remains possible at the temperature of 750 °C due to the local composition fluctuation and high local form energy (E_{local}) calculated by DFT[40]. This indicates that both $L1_2$-SRO and $L1_1$-SRO structures might be presented in CoNiV-based alloys, which was also confirmed by the ML-APT results.

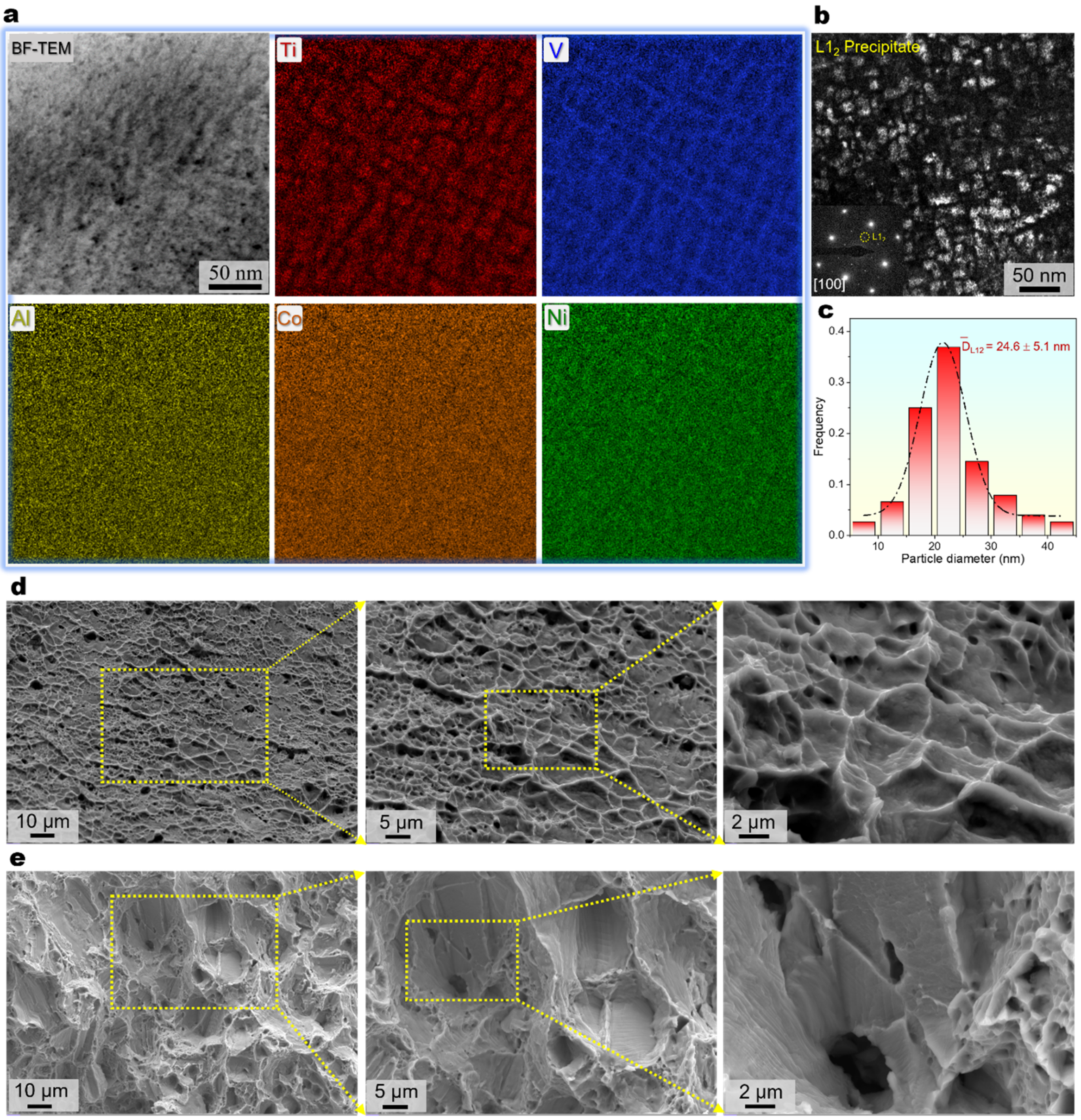

Supplementary Fig. 4 | The microstructure and fracture morphology of the CoNiV-AlTi(24h aged) sample. (a) Bright field TEM (BF-TEM) image and the corresponding EDX maps of the CoNiV-AlTi(24h aged) sample, showing the Ti/Ni enrichment and V depletion in the $L1_2$ precipitates, consistent with that of NLRO. (b) The SAED pattern and dark-field TEM (DF-TEM) image of the same sample taken from the {010} superlattice spot, showing the morphology and size of $L1_2$ precipitates. (c) The distribution of the size of $L1_2$ precipitates, determined from DF-TEM images. Fracture surface of (d) the CoNiV-AlTi sample and (e) the CoNiV-AlTi(24h aged) sample deformed at 87 K. The former sample is characterized by a dimple-typed ductile fracture, whereas the latter shows the brittle quasi-cleavage facets observed on the fracture surface.

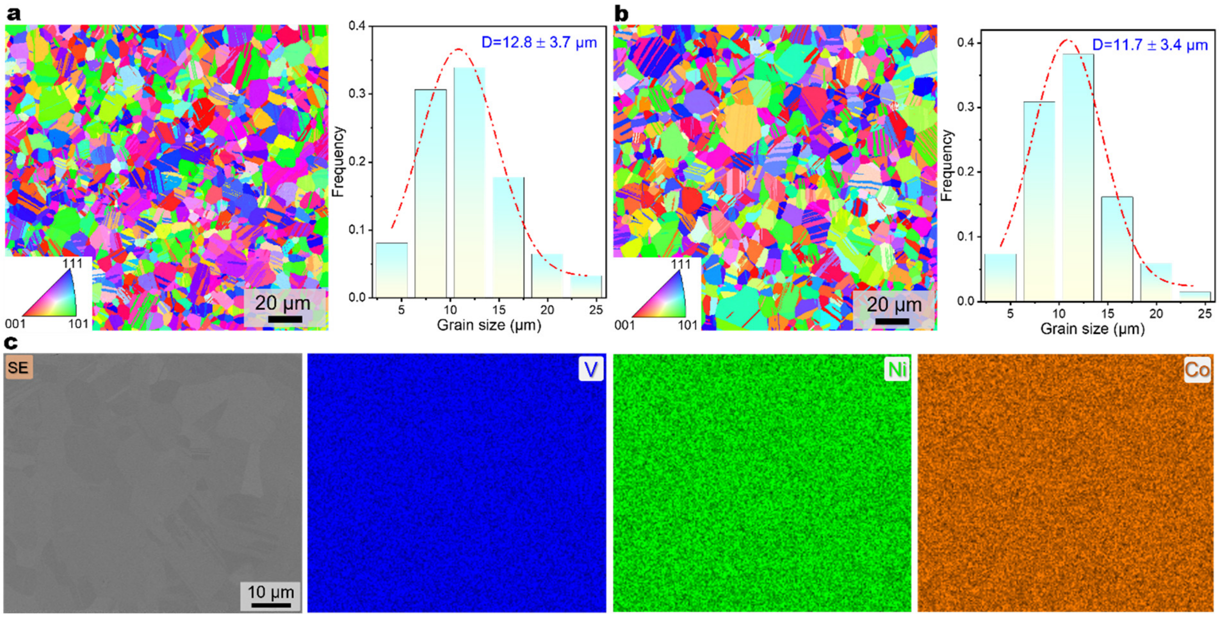

Supplementary Fig. 5 | EBSD-inverse pole figures (IPFs) of (a) the CoNiV(SS) and (b) the CoNiV(SSA) samples. Two samples with a similar grain size to the CoNiV-AlTi sample (11.2 μm), is used to study the effect of ordering. (c) SE image and EDX spectroscopy result of the CoNiV(SS) sample, indicating that the chemical composition is homogeneous after solid solution treatment.

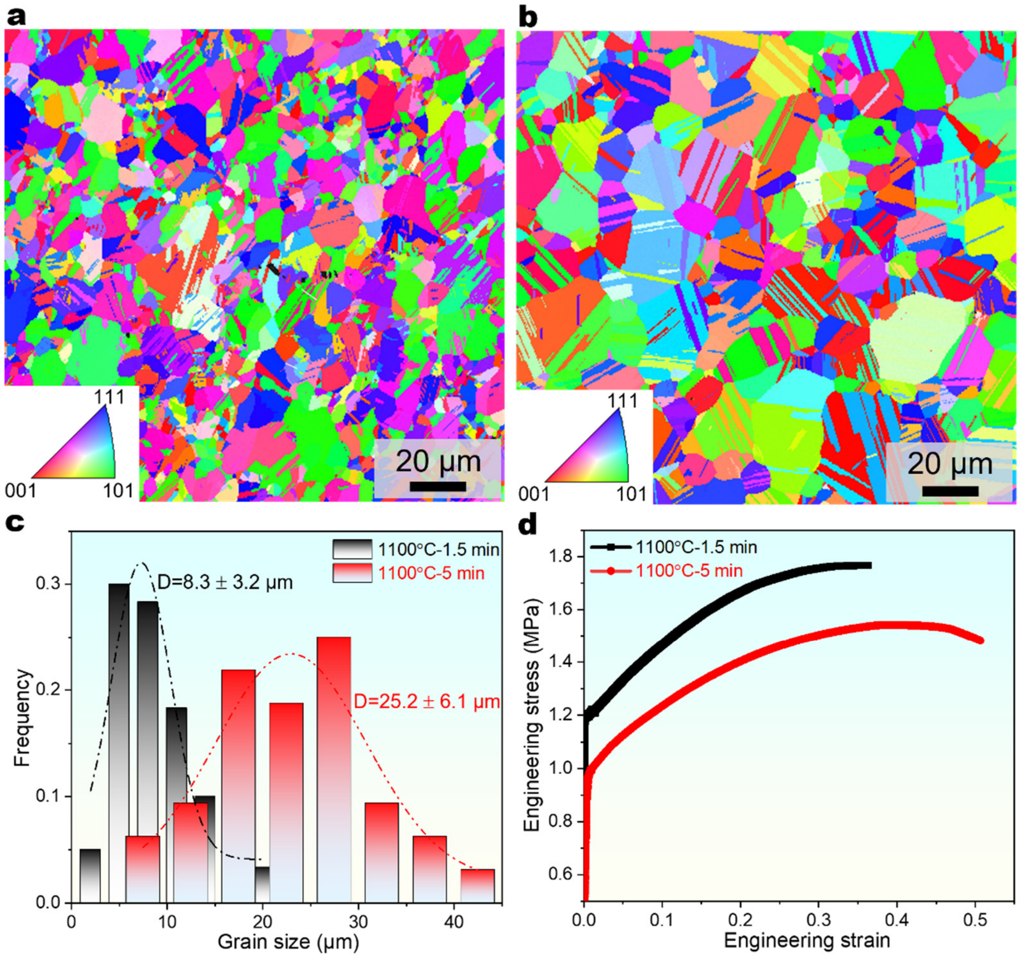

Supplementary Fig. 6 | Microstructure and cryogenic tensile property of CoNiV-AlTi sample undergoing different solid solution time at 1100 ℃. (a) and (b) EBSD-IPF images of the CoNiV-AlTi sample with different solid solution time: (a) 1.5 min and (b) 5 min, respectively. (c) Grain size distribution diagram of two samples. (d) Engineering stress-strain curves of samples with different grain sizes at 87 K.

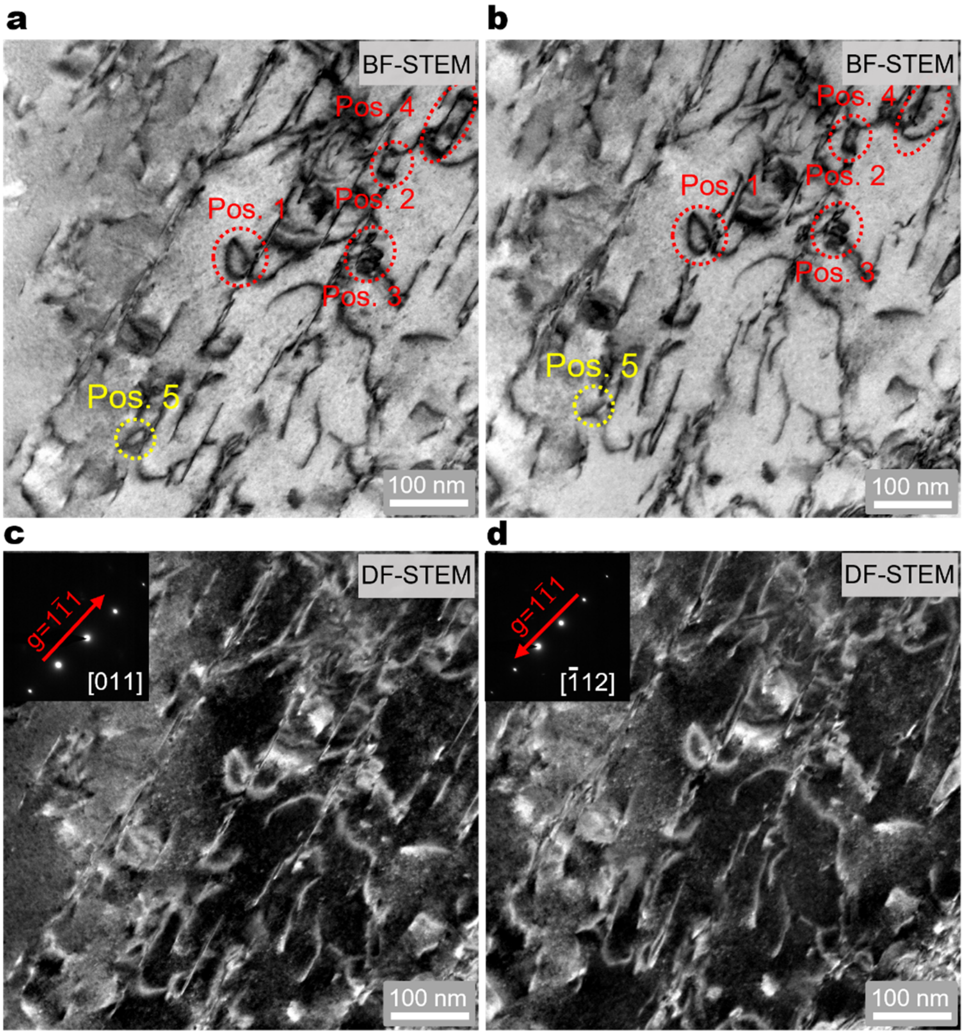

Supplementary Fig. 7 | The deformation microstructure of CoNiV-AlTi sample at the same diffraction vector under the two different zone axes. (a) and (c) BF-STEM and DF-STEM images on the $\boldsymbol{g}$ = $1\bar{1}1$ diffraction vector under the [011] zone axis. (b) and (d) BF-STEM and DF-STEM images on the $\boldsymbol{g}$ = $1\bar{1}1$ diffraction vector under the [$\bar{1}12$] zone axis. Some “dislocation loops” (Pos. 5) are artifacts due to dislocation overlapping, and they disappear when probed under another zone axis. However, the existence of a number of dislocation loops can still be confirmed (e.g., Pos. 1-4 including the one shown in the manuscript). These dislocations do not show much change under different tilting angles (or different zone axis).

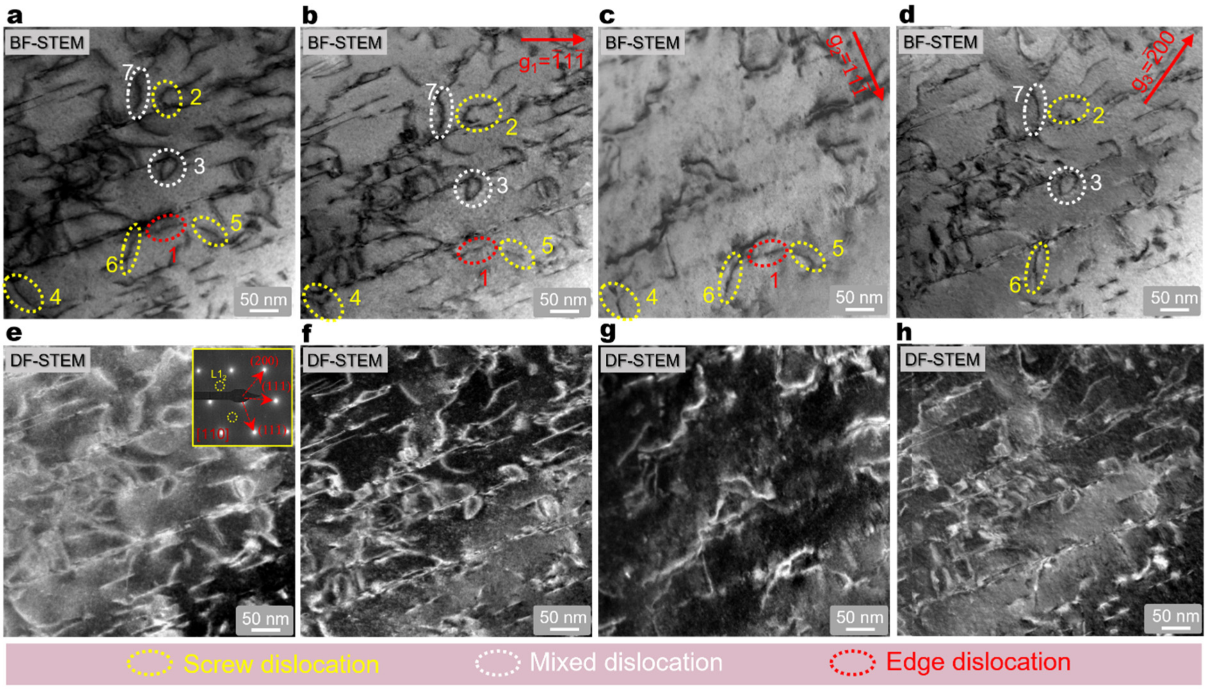

Supplementary Fig. 8 | The dislocation distribution in the CoNiV-AlTi sample. The sample cryogenically strained to 15% strain was observed at three different diffraction vectors under the [110] zone axis. (a) and (e) BF-STEM and DF-STEM images taken under the [110] zone axis. (b) and (f) BF-STEM and DF-STEM images on the $\boldsymbol{g} = \bar{1}1\bar{1}$ diffraction vector. (c) and (g) BF-STEM and DF-STEM images on the $\boldsymbol{g} = 11\bar{1}$ diffraction vector. (d) and (h) BF-STEM and DF-STEM images on the $\boldsymbol{g} = \bar{2}00$ diffraction vector. Dislocation types are concluded in Supplementary Table 4. Dislocations were probed under three different $\boldsymbol{g}$ vectors ($[1\bar{1}1]$, $[11\bar{1}]$ and $[\bar{2}00]$). Based on the $\boldsymbol{g}\cdot\boldsymbol{b}$ criterion, i.e., dislocations become invisible when $\boldsymbol{g}\cdot\boldsymbol{b} = 0$, the Burgers vectors of these dislocations can be determined. The basis for determining the dislocation type is that the edge dislocation line is perpendicular to its Burgers vector while the screw dislocation line is parallel to its Burgers vector. Above results reveal the cross-slip of high-density screw dislocations emitted from planar-slip bands in the CoNiV-AlTi sample.

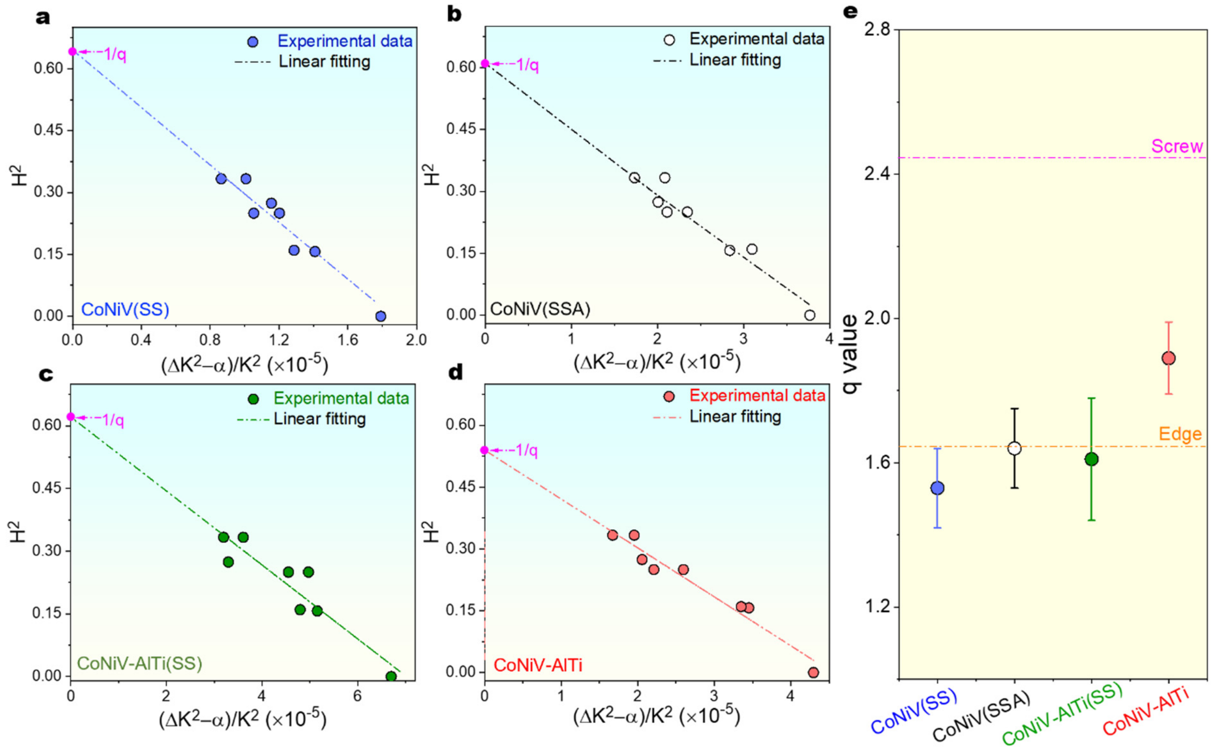

Supplementary Fig. 9 | Qualitative assessment of dislocation types for four samples. The $(\Delta K - \alpha)^2/K^2$ versus H^2 plot of (a) the solid-solution treated CoNiV(SS) sample, (b) the solid-solution and aged CoNiV(SSA) sample containing only SRO, (c) solid-solution treated CoNiV-AlTi(SS) sample and (d) solid-solution and aged CoNiV-AlTi sample containing both SRO and NLRO. The samples were cryogenically (87 K) strained to 15%. The data were analyzed on the basis of neutron diffraction data. (e) Determined dislocation characteristic parameters, q, for four samples. Results show that there is no significant difference between solid-solution treated CoNiV(SS) and CoNiV-AlTi(SS) samples, whereas a higher q value is observed for the aged CoNiV-AlTi sample containing dual-scale ordering.

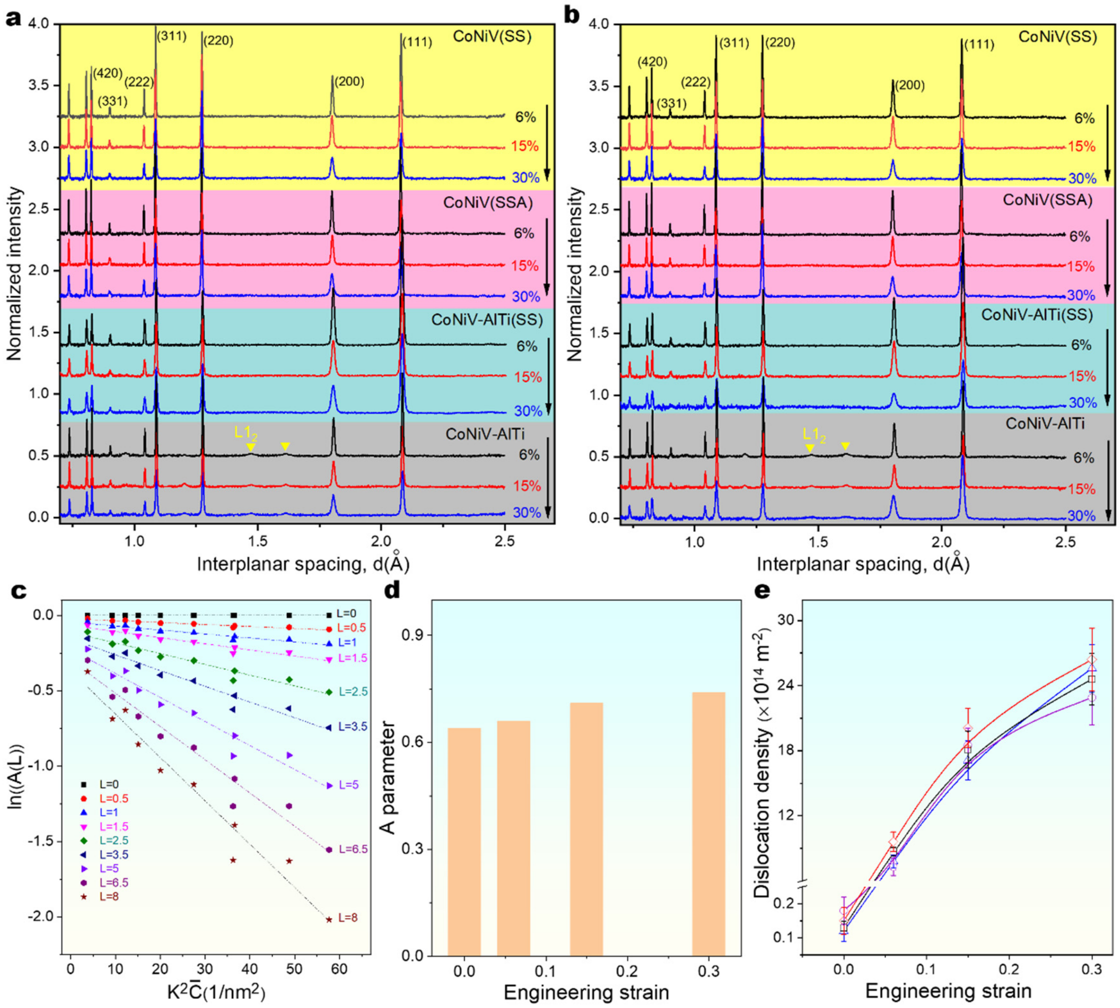

Supplementary Fig. 10 | Neutron diffraction patterns and the calculation of dislocation density. (a) Neutron diffraction profiles of the CoNiV(SS), CoNiV(SSA), CoNiV-AlTi(SS) and CoNiV-AlTi samples deformed at different strains at 293 K. The background is taken as the base in order to better display the peaks or refinement results in all neutron diffraction patterns. (b) Neutron diffraction profiles of the CoNiV(SS), CoNiV(SSA), CoNiV-AlTi(SS) and CoNiV-AlTi samples deformed at different strains at 87 K. (c) Modified Warren-Averbach plot obtained from the peaks in the neutron profile of the CoNiV(SS) sample with a strain of 6% at 293 K. (d) The calculation of the *A* value as a function of the engineering strain of the CoNiV(SS) sample with a strain of 6% at 293 K. (e) Dislocation density versus engineering strain of the investigated samples deformed at 293 K, which indicates that there is no significant difference in dislocation evolution among four samples at this temperature.

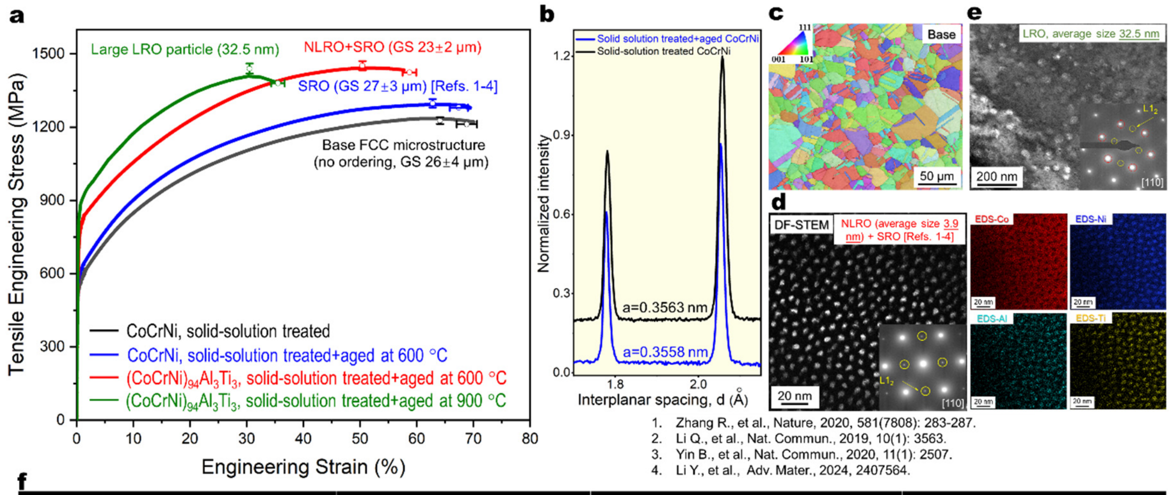

f

Materials (at.%)	Ordering type	Solution treatment	Aging treatment
CoCrNi	Base	1100 °C for 6 min	-
CoCrNi	SRO	1100 °C for 6 min	600 °C for 3h
$(CoCrNi)_{94}Al_3Ti_3$	NLRO+SRO	1100 °C for 6 min	600 °C for 3h
$(CoCrNi)_{94}Al_3Ti_3$	LRO	1100 °C for 6 min	900 °C for 3h

Supplementary Fig. 11 | Cryogenic (87 K) mechanical properties and representative microstructure of the CoCrNi-based alloys with different ordering states. (a) Engineering stress-strain curve of the solid-solution treated and 600 °C aged $(CoCrNi)_{94}Al_3Ti_3$ sample with a dual-scale ordering, in comparison with the reference solid-solution treated CoCrNi sample, the solid-solution treated and aged CoCrNi sample and the high-temperature (900 °C) aged $(CoCrNi)_{94}Al_3Ti_3$ sample containing larger $L1_2$ precipitates. The testing temperature was 87 K. (b) Neutron diffraction patterns of solid-solution treated CoCrNi and solid-solution treated + aged CoCrNi samples, displaying detailed differences in the lattice constants of the solid-solution treated and aged samples (0.3563 nm and 0.3558 nm, respectively), which indicates that the aging treatment leads to higher possibility of SRO in combination with the slightly enhanced strength. (c) EBSD-IPF image of solid-solution treated CoCrNi alloy. The grain size (GS) is similar among these samples. (d) DF-STEM and EDS mapping of the 600 °C aged $(CoCrNi)_{94}Al_3Ti_3$ sample, showing the formation of NLRO with an average diameter of 3.9 nm. (e) The BF-TEM image of the high-temperature (900 °C) aged $(CoCrNi)_{94}Al_3Ti_3$ sample, showing the formation of larger $L1_2$ precipitates with an average diameter of 32.5 nm. (f) The relationship among chemical composition, heat treatment and ordering parameters in CoCrNi systems. Before heat treatment, these samples were synthesized by arc melting, homogenization (1100 °C for 24 h) and cold rolling (80%).

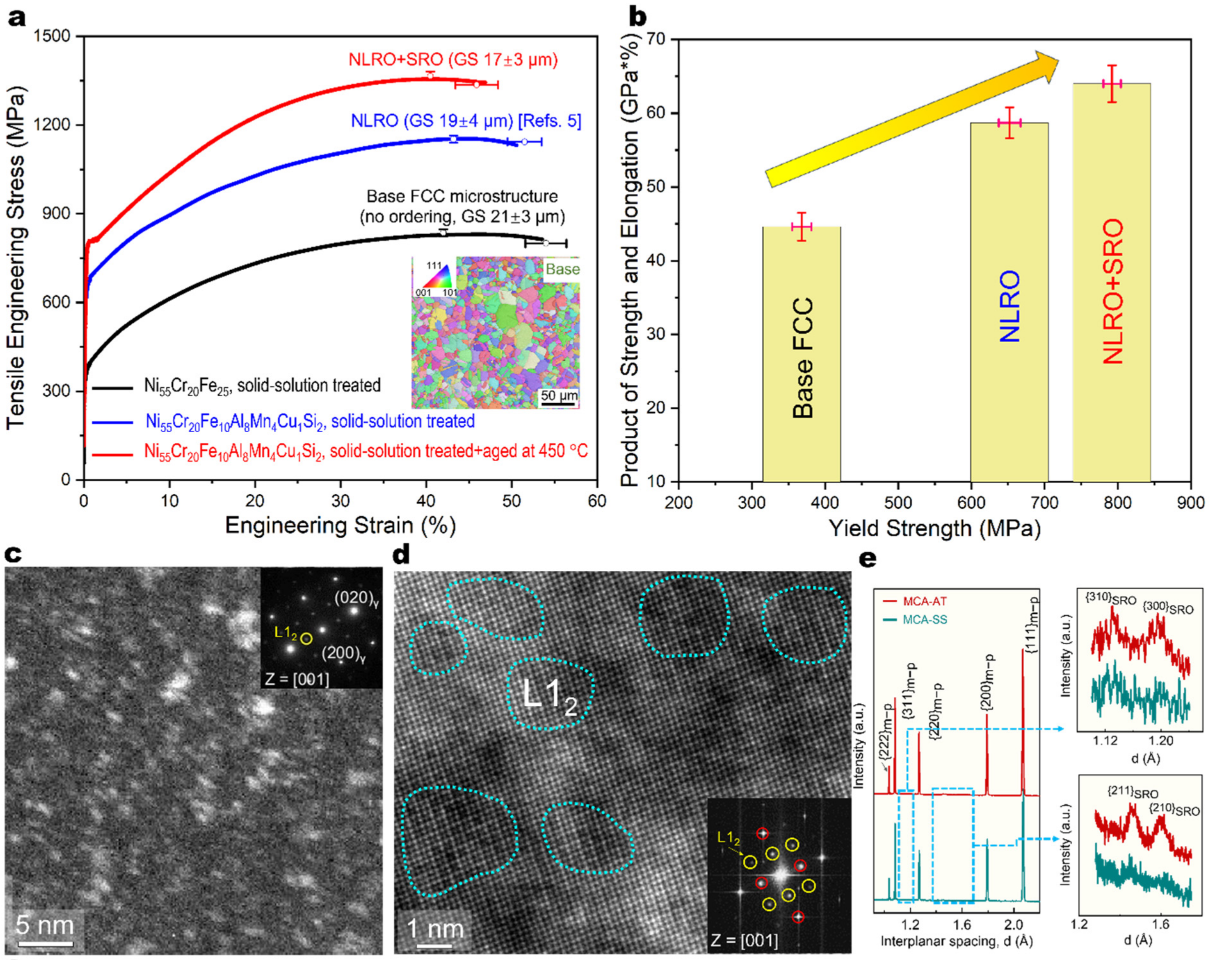

f

Materials (at.%)	Ordering	Solution treatment	Aging treatment
$Ni_{55}Cr_{20}Fe_{25}$	Base	950 °C for 3 min	-
$Ni_{55}Cr_{20}Fe_{10}Al_8Mn_4Cu_1Si_2$	NLRO	950 °C for 3 min	-
$Ni_{55}Cr_{20}Fe_{10}Al_8Mn_4Cu_1Si_2$	NLRO+SRO	950 °C for 3 min	450 °C for 72 h

Supplementary Fig. 12 | Cryogenic (87 K) mechanical properties and representative microstructure of the NiCrFe-based alloys with different ordering states. (a) Engineering stress-strain curve of the annealed and aged $Ni_{55}Cr_{20}Fe_{10}Al_8Mn_4Cu_1Si_2$ sample (MCA-AT) with a dual-scale ordering structure, in comparison with the annealed $Ni_{55}Cr_{20}Fe_{25}$ base sample and annealed $Ni_{55}Cr_{20}Fe_{10}Al_8Mn_4Cu_1Si_2$ sample (MCA-SS). The test temperature was 87 K. The inset is EBSD-IPF of the base sample. The GS is similar among the three samples. (b) The product of tensile strength and total elongation as a function of the yield strength of the three samples. (c) DF-TEM image of the aged $Ni_{55}Cr_{20}Fe_{10}Al_8Mn_4Cu_1Si_2$ sample, showing the morphology of nanoscale $L1_2$ precipitates[41]. (d) HR-STEM image with the [001] zone axis and the corresponding Fast Fourier Transform pattern, showing the size of the $L1_2$ nanoprecipitates[41]. (e) Neutron diffraction patterns of the annealed and aged $Ni_{55}Cr_{20}Fe_{10}Al_8Mn_4Cu_1Si_2$ sample and the reference annealed $Ni_{55}Cr_{20}Fe_{10}Al_8Mn_4Cu_1Si_2$ sample[41], which indicates the formation of SRO in the former sample. (f) The relationship among chemical composition, heat treatment and ordering parameters in NiCrFe-based materials. Before heat treatment, there samples were synthesized by arc melting, hot-rolling, homogenization (1100 °C for 2 h) and cold rolling (80%).

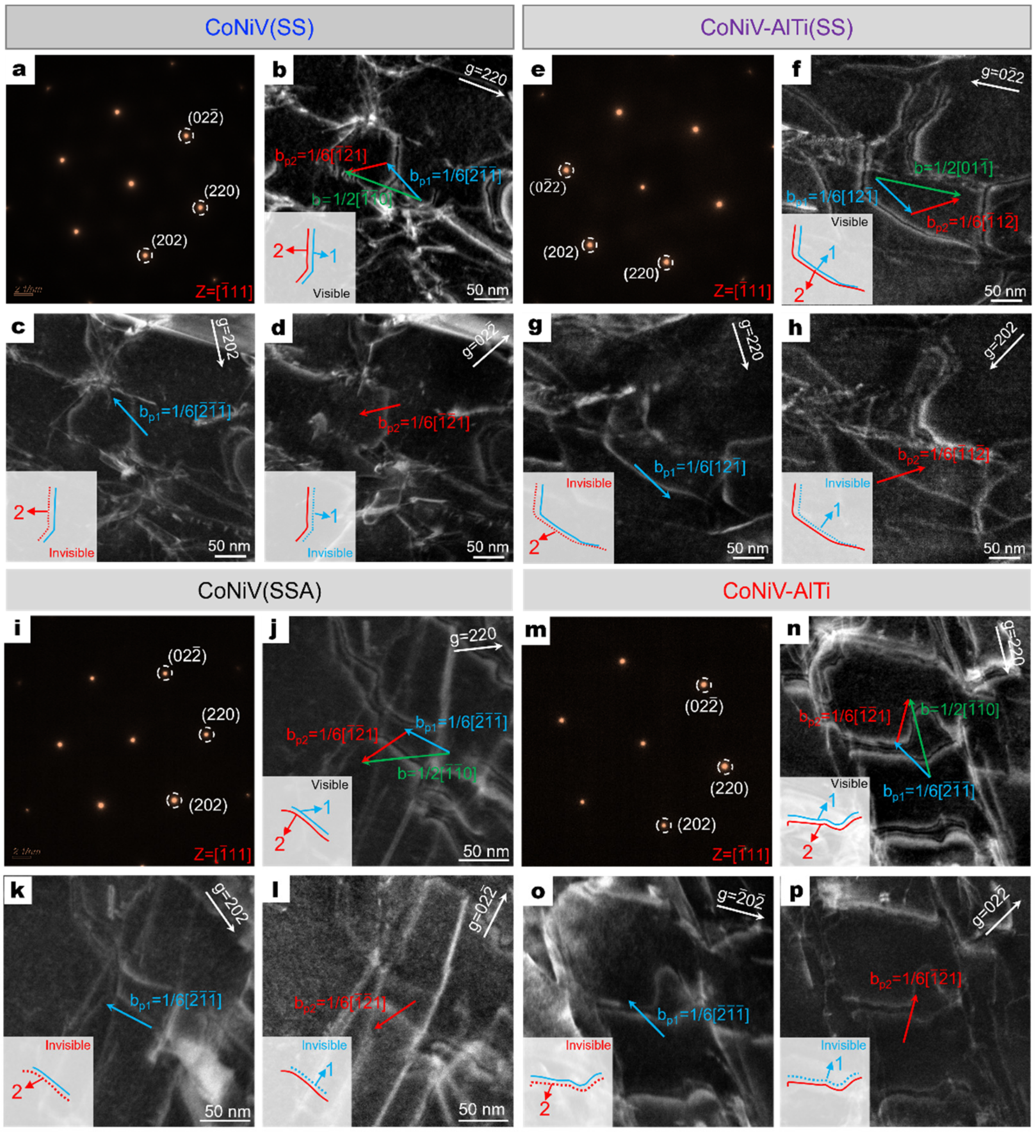

Supplementary Fig. 13 | Detailed g·b analysis of partial dislocations in the cryogenically (87 K) strained (6%) samples and the calculation of SFE. (a-d) Ordering-free CoNiV(SS) sample, (e-h) ordering-free CoNiV-AlTi(SS) sample, (i-l) SRO-containing CoNiV(SSA) sample, and (m-p) dual-scale ordering-containing CoNiV-AlTi sample. (a), (e), (i) and (m) Selected area electron diffraction (SAED) pattern along the zone axis of [$\bar{1}11$]. (b-d), (f-h), (j-l) and (n-p) Weak-beam STEM images corresponding three different *g* vectors. The left-bottom schematic diagrams represent the visible or invisible condition of dislocation under the corresponding g vector. The Burgers vectors of the visible dislocations are noted on the images.

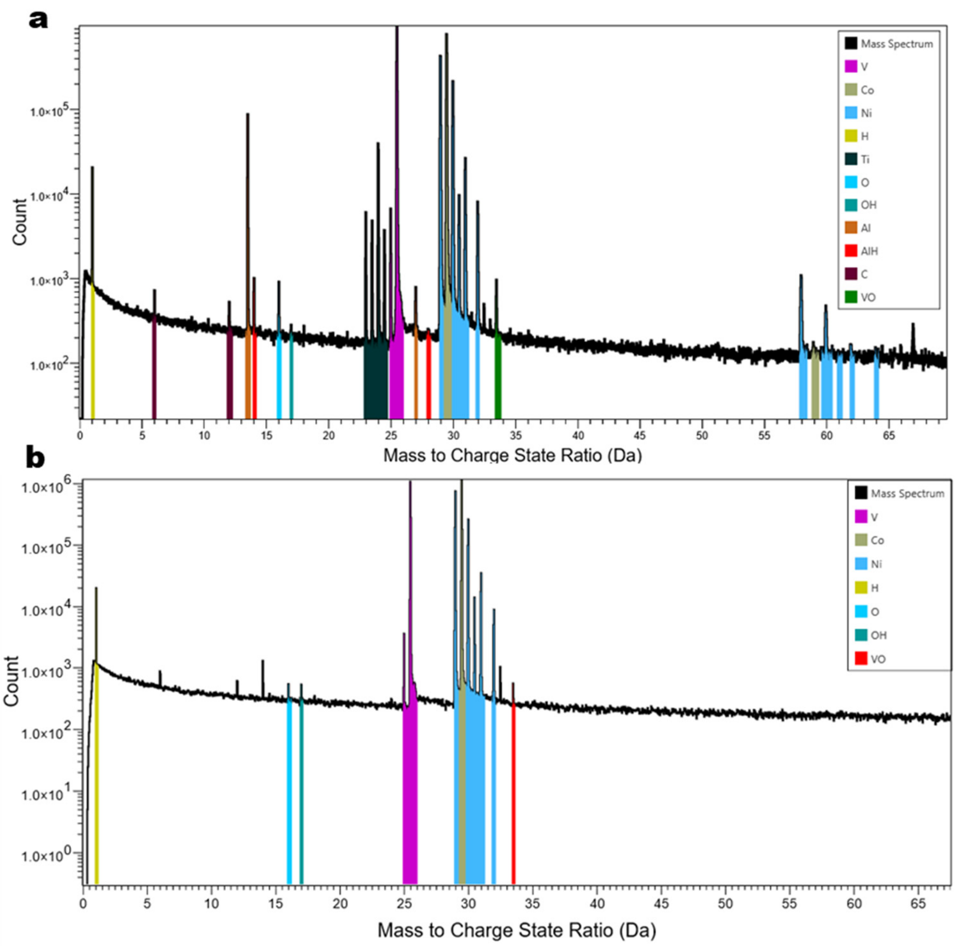

Supplementary Fig. 14 | Typical APT time-of-flight mass spectrum with identified ions. (a) the CoNiV-AlTi sample and (b) the CoNiV(SSA) sample.

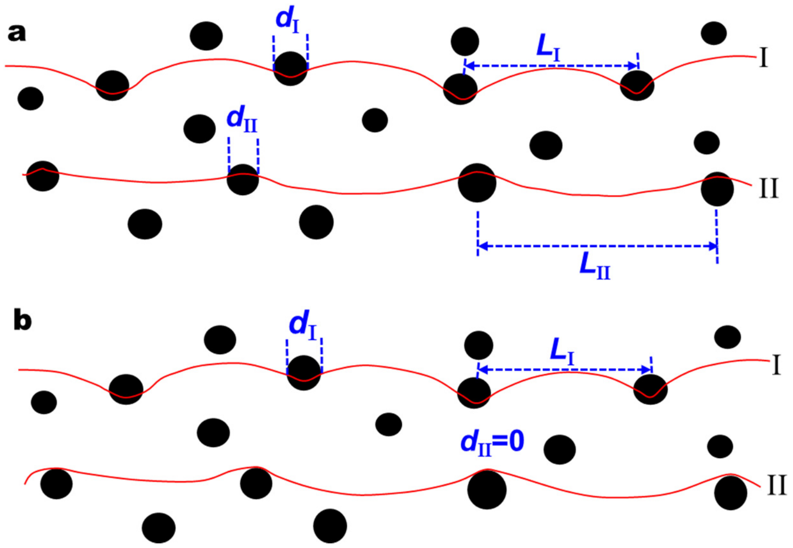

Supplementary Fig. 15 | Schematic diagram of ordered domains sheared by a pair of dislocations. In (a) d_{II} is finite, while in (b) $d_{II} = 0$.

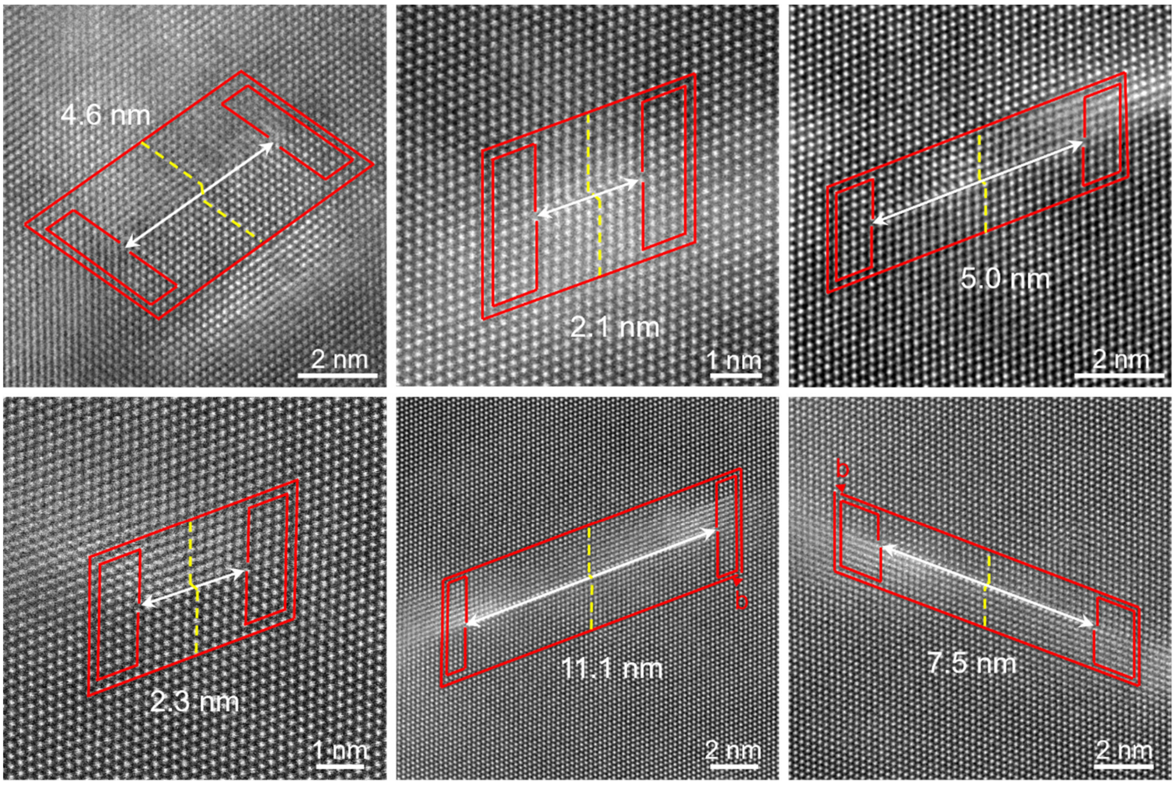

Supplementary Fig. 16 | Typical atomic-scale resolution HAADF-STEM images of the CoNiV(SS) sample. The sample undergoes at a global sample strain of 6% at the cryogenic temperature (87 K), showing dissociations of full dislocations into Shockley partials with various SF widths. At least 20 pairs of partial dislocations from dissociations of full dislocations were measured and quantified.

Supplementary Table 1 | Thermal and physical parameters of elements. Atomic radius, shear modulus, electronegativity and vacancy electron concentration (VEC) of the solute elements in the CoNiV alloy[42-46]

Element	Atomic radius (pm)[42]	VEC[43]	Crystal structure	Electronegativity[44]	Shear modulus (GPa)[45]	Mixing enthalpy (kJ/mol)[46]	
						Ni	V
Co	125	9	FCC (> 422 °C), Hexagonal (< 422 °C)	1.63	75	0	-14
Ni	124	10	FCC	1.91	76	-	-18
V	134	5	BCC	1.63	47	-	-

From a thermodynamic point of view, the driving force for the formation of SRO in CoNiV might be originated from multiple aspects, including the fluctuation in the local strain[47], bonding state[48,49], electronic and magnetic interactions[50,51]. The fluctuation of local strain energy in MEAs/HEAs is normally pronounced since they generally possess severe lattice distortion. The enlargement in the atomic size mismatch among solute elements would raise the variation of lattice distortion and consequently the strain energy. Therefore, the large atomic size mismatch between V and Co/Ni promotes the formation of SRO. The bonding energy, which can be simply reflected by the negative mixing enthalpy between different elements, is another important factor for the formation of SRO. The mixing enthalpy between Ni or Co and V is -18 kJ/mol and -14 kJ/mol, respectively, indicating a strong affinity between V and Co/Ni, which promotes the formation of SRO in CoNiV-based alloys.

Supplementary Table 2 | SFE, elastic and mechanical properties of investigated samples

**Material properties**	**Parameters**	**CoNiV (SS)**	**CoNiV (SSA)**	**CoNiV-AlTi (SS)**	**CoNiV-AlTi**
Elastic properties	Poisson Ratio	0.31	0.31	0.30	0.30
	Young modulus (GPa)	191	190	188	189
	Shear modulus (GPa)	72.9	72.1	72.3	72.6
Mechanical properties at 87 K	σ_{YS} (MPa)	818±23	958±18	845±21	1152±24
	σ_{UTS} (MPa)	1379±29	1494±24	1410±32	1768±28
	TE (%)	52.9±1.5	48.1±1.4	51.8±2.1	42.6±2.4
	Fracture toughness (MPa·$m^{1/2}$)	324.0±4.8	299.5±11.9	329.5±6.7	338.4±8.2
SFE at 87 K	mJ/m^2	52.3±6.5	60.2±8.5	48.4±8.2	62.6±7.9

σ_{YS}: Yield strength, σ_{UTS}: Ultimate tensile strength, TE: Total elongation.

Supplementary Table 3 | Details of the data presented in Fig. 2b

Materials classification	Test temperature	Materials composition	Mechanical properties			Ref.
			σ_{YS} (MPa)	σ_{UTS} (MPa)	TE (%)	
Single-phase FCC MEA/HEAs (at.%)	77K	CoCrFeNi	590	1070	78	52
	77K	CoCrNi	860	1305	46.9	53
	77K	CoCrNi	520	1200	78	54
	77K	CoCrNi	657	1311	90	55
	77K	$CoCrNiAl_6Ta_2$	786	1351	90	56
	77K	CrFeCoNiPd	660	1010	69	11
	77K	CrFeCoNiPd	880	1220	52	11
	77K	CrMnFeCoNi	670	1270	72	11
	77K	CrMnFeCoNi	380	880	105	11
	77K	$Cr_{26}Mn_{20}Fe_{20}Co_{20}Ni_{14}$	500	1100	62	57
FCC+$L1_{2(p)}$ MEA/HEAs (at.%)	77K	$Co_{40}Cr_{20}Ni_{30}Al_5Ti_5$	2022	2309	21.2	58
	77K	$Co_{35}Ni_{35}Fe_{20}Al_5Ti_5$	930	1320	21	59
	77K	$Co_{35}Ni_{35}Fe_{20}Al_5Ti_5$	1020	1460	26.4	59
	77K	$Co_{34.46}Cr_{32.12}Ni_{27.42}Al_3Ti_3$	730	1340	67	60
	77K	$Co_{34.46}Cr_{32.12}Ni_{27.42}Al_3Ti_3$	980	1680	35.2	60
FCC+B2 MEA/HEAs (at.%)	77K	$Al_{0.3}CoCrFeNi$	1320	1600	17.5	61
	77K	$Al_{0.3}CoCrFeNi$	1300	1500	16.4	61
	77K	$Al_{0.3}CoCrFeNi$	1300	1500	18	61
	77K	$Al_{0.5}CoCrFeMnNi$	796	1329	30.5	62
	77K	$AlCoCrFeNi_{2.0}$	715	952	3.7	63
	77K	$AlCoCrFeNi_{2.1}$	690	1051	6.7	63
	77K	$AlCoCrFeNi_{2.2}$	705	1151	9.3	63
TRIP HEA/MEAs (at.%)	77K	$Fe_{60}Co_{15}Ni_{15}Cr_{10}$	600	1020	128	64
	77K	$Fe_{60}Co_{15}Ni_{15}Cr_{10}$	660	1500	85	64
	77K	$Fe_{60}Co_{15}Ni_{15}Cr_{10}$	600	1180	118	64
	77K	$Fe_{49}Mn_{30}Co_{10}Cr_{10}$	398	1323	53	65
	77K	$Fe_{49}Mn_{30}Co_{10}Cr_{10}N_{1.8}$	1206	1620	11	65
	77K	$Fe_{49}Mn_{30}Co_{10}Cr_1$	1078	1630	33.5	65
	77K	$Fe_{65}Ni_{15}Co_8Mn_8Ti_3Si$	1305	1800	45	66
	77K	$Co_{17.5}Cr_{12.5}Fe_{55}Ni_{10}Mo_4C_1$	984	1762	54.7	67
	77K	$Co_{17.5}Cr_{12.5}Fe_{55}Ni_{10}Mo_3C_2$	1010	1978	53.3	67
	77K	$V_{10}Cr_{10}Co_{30}Fe_{40}Ni_{10}$	706	1256	64.4	68
	77K	$V_{10}Cr_{10}Co_{30}Fe_{45}Ni_5$	740	1358	71.2	68
	77K	$V_{10}Cr_{10}Co_{30}Fe_{50}$	520	1990	38.8	68
	77K	$(Fe_{40}Mn_{40}Co_{10}Cr_{10})_{96.7}C_{3.3}$	551	1038	21	69
	77K	$(Fe_{40}Mn_{40}Co_{10}Cr_{10})_{96.7}C_{3.3}Ti$	987	1377	51.5	69
	77K	$Fe_{50}Co_{25}Ni_{10}Al_5Ti_5Mo_5$	1305	2340	16	70
Fe-based alloys (wt.%)	77K	19Mn-0.45C	680	1377	25.4	71
	77K	22Mn-0.45C	646	1509	53.5	71
	77K	19Mn-0.45C-2Al	759	1355	40.7	71
	77K	22Mn-0.45C-2Al	811	1403	67.2	71
	77K	20Mn–4Al-0.3C	698.6	1196.5	77.4	72
	77K	27Mn–4Al-0.3C	729.8	1173.0	68.3	72
	77K	18Cr-Ni-Mn-Si	401	1,417	13	73
	77K	9Ni	980	1,201	24	73
	77K	Fe-8.53Ni-0.002C	794	851	37.3	74

	77K	Fe-8.53Ni-0.002C	700	1200	67	74
	110K	Invar steel	600	870	50	75
	77K	Fe-25Mn-4Cr-0.5C	800	1380	58	76
	77K	Fe-25Cr-7Ni- 3Mo	1020	1280	45	77
BCC HEA alloys (at.%)	77K	TiZrHfNbTa	1547	1549	20.8	78
	77K	$FeCoCrNiMo_{0.2}$	637	1212	71.2	79
Al-based alloys	77K	Al-Cu-Mn alloy	210	415	42	80
	77K	1575C Al	260	470	16	81
	77K	1575C Al	335	555	34	81
	77K	AA2195	600	680	7	82
	77K	AA2195	615	670	7	82
	77K	AA5083	320	420	25	83
Ni-based alloys	100K	Haynes 282	700	1200	19	84
	77K	GH3536	593	1198	76	85
	77K	Alloy 625	550	1134	89.4	86
	77K	Haynes 282	750	1350	15	84
	77K	INCONEL 718	1327	1640	12.5	87
Ti-based alloys	77K	Ti-3Al-3Mo-3Zr alloy	1290	1310	10.5	88
	77K	Ti-15Mo-2Al	1055	1536	22	89
	77K	Ti-5Al-1V-1Sn-1Zr-0.8Mo	1252.3	1326.3	25	90
	77K	Ti-6Al-4V	1600	1680	10.5	91

Supplementary Table 4 | The Burgers vector of dislocations using the *g*·*b* criterion, corresponding to Supplementary Fig. 8

Screw dislocation	***g***-vector			Dislocation type	***b***
	$[1\bar{1}1]$	$[11\bar{1}]$	$[\bar{2}00]$		
1	√	√	×	Edge	$1/2[01\bar{1}]$
2	√	×	√	Screw	$1/2[1\bar{1}0]$
3	√	×	√	Mixed	$1/2[1\bar{1}0]$
4	√	√	×	Screw	$1/2[01\bar{1}]$
5	√	√	×	Screw	$1/2[01\bar{1}]$
6	×	√	√	Screw	$1/2[10\bar{1}]$
7	√	×	√	Mixed	$1/2[1\bar{1}0]$

* '√' means visible, while '×' means invisible in TEM images shown in Supplementary Fig. 8.

Supplementary Table 5 | Details of the data presented in Extended Data Fig. 3b

Classification	Test temperature	Materials	Mechanical properties		Ref.
			σ_{YS} (MPa)	K_{Ic} (MPa • $m^{1/2}$)	
MEAs & HEAs	77K	CoCrNi	657	273.3	55
	77K	CoCrNi	944	176.3	92
	77K	$V_{10}Fe_{45}Cr_{10}Co_{30}Ni_5$	687	237	93
	77K	$V_{10}Fe_{45}Cr_{10}Co_{20}Ni_{15}$	470	232	93
	77K	$Si_8V_2Fe_{45}Cr_{10}Mn_5Co_3$	457	129	94
	77K	CoCrFeNiMn	759	219	15
	77K	$Al_{0.5}CrCoFeNi$	1376	51	13
	77K	$Al_{0.5}CrCoFeNi$	942	291	13
Fe-based alloys	77K	9Ni alloy	1137	177	95
	77K	9Ni alloy	1058	179	95
	77K	9Ni alloy	998	189	95
	77K	5Ni alloy	1118	99	95
	77K	5Ni alloy	995	102	95
	77K	5Ni alloy	855	108	95
	77K	5Ni alloy	1113	190	95
	77K	5Ni alloy	859	130	95
	77K	12Cr-12Ni-10Mn-5Mo	~850	~340	96
	77K	12Cr-12Ni-10Mn-5Mo	~850	~450	96
	77K	12Cr-12Ni-10Mn-5Mo	~850	~500	96
Ti-based alloys	123K	Ti6Al4V	1300	54.36	97
	77K	Ti6Al4V	~1500	~35	98
	77K	Ti6Al4V	~1400	~41	98
	77K	Ti6Al4V	~1380	~58	98
	77K	Ti-5AI-2.5Sn	1203	114.3	99
	77K	Ti-5AI-2.5Sn	1215	131.1	99
	77K	Ti6Al4V	1480	46	100
	77K	Ti6Al4V	1750	33	100
	77K	Ti6Al4V	1720	28	100
Al-based alloys	77K	2090- T81	589	51	101
	77K	8091-T8	574	38	101
	77K	2090- T8	483	44	101
	77K	2090-T83	568	30	101
	77K	2091-T351	442	41	101
	77K	8091-T351	382	28	101

Supplementary References:

1. He J. Y., Wang H., Huang H. L., *et al.* A precipitation-hardened high-entropy alloy with outstanding tensile properties. *Acta Mater*. **102**, 187-196 (2016).
2. Zhang R. P., Zhao S. T., Ophus C., *et al.* Direct imaging of short-range order and its impact on deformation in Ti-6Al. *Sc. Adv*. **5**(12), x2799 (2019).
3. Ming K. S., Bi X. F., Wang J. Realizing strength-ductility combination of coarse-grained $Al_{0.2}Co_{1.5}CrFeNi_{1.5}Ti_{0.3}$ alloy via nano-sized, coherent precipitates. *Int. J. Plasticity* **100**, 177-191 (2018).
4. Ardell A. J. Precipitation Hardening. *Metall. Trans. A* **16**A, 1985-2131 (1985).
5. Zhao Y. L., Yang T., Tong Y., *et al.* Heterogeneous precipitation behavior and stacking-fault-mediated deformation in a CoCrNi-based medium-entropy alloy. *Acta Mater.* **138**, 72-82 (2017).
6. Jiang L., Yang H., Yee J. K., *et al.* Toughening of aluminum matrix nanocomposites via spatial arrays of boron carbide spherical nanoparticles. *Acta Mater.* **103**, 128-140 (2016).
7. Seidman D. N., Marquis E. A. & Dunand D. C. Precipitation strengthening at ambient and elevated temperatures of heat-treatable Al(Sc) alloys. *Acta Mater.* **50**, 4021-4035 (2002).
8. Wen H., Topping T. D., Isheim D., *et al.* Strengthening mechanisms in a high-strength bulk nanostructured Cu-Zn-Al alloy processed via cryomilling and spark plasma sintering. *Acta Mater.* **61**, 2769-2782 (2013).
9. Du X. H., Li W. P. Chang H. T., *et al.* Dual heterogeneous structures lead to ultrahigh strength and uniform ductility in a Co-Cr-Ni medium-entropy alloy. *Nat. Commun.* **11**, 2390 (2020).
10. Lu W., Gong J., Huang B., *et al.* Optimizing precipitation hardening in a L12-strengthened medium-entropy alloy via tuning the anti-phase boundary energy. *Scr. Mater.* **245**, 116045 (2024).
11. Ding Q., Zhang Y., Chen X., *et al.* Tuning element distribution, structure and properties by composition in high-entropy alloys. *Nature* **574**, 223-227, (2019).
12. Zhang R., Zhao S., Ding J., *et al.* Short-range order and its impact on the CrCoNi medium-entropy alloy. *Nature* **581**(7808), 283-287 (2020).
13. Kumar P., Huang S., Cook D., *et al.* A strong fracture-resistant high-entropy alloy with nano-bridged honeycomb microstructure intrinsically toughened by 3D-printing. *Nat. Commun.* **15**(1) 841 (2024).
14. Liu D., Yu Q., Kabra S., *et al.* Exceptional fracture toughness of CrCoNi-based medium- and high-entropy alloys at 20 kelvin. *Science* **378**, 978-983, (2022).
15. Gludovatz B., Hohenwarter A., Catoor D., *et al.* A fracture-resistant high-entropy alloy for cryogenic applications. *Science* **345**(6201), 1153-1158 (2014).
16. Miao J. S., Slone C., Dasari S., *et al.* Ordering effects on deformation substructures and strain hardening behavior of a CrCoNi based medium entropy alloy. *Acta Mater.* **210**, 116829 (2021).
17. Laplanche G., Kostka A., Reinhart C., *et al.* Reasons for the superior mechanical properties of medium-entropy CrCoNi compared to high-entropy CrMnFeCoNi. *Acta Mater.* **128**, 292-303, (2017).
18. Wang F., Song M., Elkot M., *et al.* Shearing brittle intermetallics enhances cryogenic strength and ductility of steels. *Science* **384**, 1017-1022 (2024).
19. Chou T. H., Li W. P., Zhu L. Y., *et al.* Critical impacts of thermodynamic instability and short-range order on deformation mechanisms of VCoNi medium-entropy alloy. *Acta Mater*. **277**, 120190 (2024).
20. Zhou D., Chen Z., Ehara K., *et al.* Effects of annealing on hardness, yield strength and dislocation structure in single crystals of the equiatomic Cr-Mn-Fe-Co-Ni high entropy alloy. *Scripta Mater*. **191**, 173-178 (2021).
21. COCKAYNE, D. J. H. The Weak-Beam Technnique As Applied to Dissociation Measurements. *Le J. Phys. Colloq*. **35**, C7-141-C7-148 (1974).

22. Schaffer, B. in Transmission Electron Microscopy. Ch. Chapter 6, 167-196 (2016).
23. Li Y., Colnaghi T., Gong Y. L., *et al.* Machine Learning-Enabled Tomographic Imaging of Chemical Short-Range Atomic Ordering. *Adv. Mater.* **36**, 2407564 (2024).
24. Li Y., Wei Y., Wang Z., *et al.* Quantitative three-dimensional imaging of chemical short-range order via machine learning enhanced atom probe tomography. *Nat. Commun.* **14**(1), 7410 (2023).
25. E08 Committee, E1820-13 Standard Test Method for Measurement of Fracture Toughness (ASTM International, 2013).
26. Ungár T., Révész Á., Borbély A. Dislocations and grain size in electrodeposited nanocrystalline Ni determined by the Modified Williamson-Hall and Warren-Averbach procedures. *J. Appl. Crystallogr.* **31**(4), 554-558 (1998).
27. Liu L., Yu Q., Wang Z., *et al.* Making ultrastrong steel tough by grain-boundary delamination. *Science* **368**(6497), 1347-1352 (2020).
28. Ungár T., Dragomir I., Révész Á., *et al.* The contrast factors of dislocations in cubic crystals: the dislocation model of strain anisotropy in practice. *J. Appl. Crystallogr.* **32**(5), 992-1002 (1999).
29. Naeem M., He H., Harjo S., *et al.* Temperature-dependent hardening contributions in CrFeCoNi high-entropy alloy. *Acta Mater.* **221**, 117371 (2021).
30. Qiu S., Zheng G. P., Jiao Z. B. Alloying effects on phase stability, mechanical properties, and deformation behavior of CoCrNi-based medium-entropy alloys at low temperatures. *Intermetallics* **140**, 107399 (2022).
31. Tang L., Jiang F. Q., Wróbel J. S., *et al.* In situ neutron diffraction unravels deformation mechanisms of a strong and ductile FeCrNi medium entropy alloy. *J. Mater. Sci. Technol.* **116**, 103-120 (2022).
32. Wang Y., Liu B., Yan K., *et al.* Probing deformation mechanisms of a FeCoCrNi high-entropy alloy at 293 and 77 K using in situ neutron diffraction. *Acta Mater.* **154**, 79-89 (2018).
33. Ren J., Zhang Y., Zhao D., *et al.* Strong yet ductile nanolamellar high-entropy alloys by additive manufacturing. *Nature* **608**, 62-68 (2022).
34. Amalia L., Li Y., Bei H., *et al.* Copper effects on the microstructures and deformation mechanisms of CoCrFeNi high entropy alloys. *Appl. Phys. Lett.* **124**(14) (2024).
35. HajyAkbary F., Sietsma J., Böttger A., *et al.* An improved X-ray diffraction analysis method to characterize dislocation density in lath martensitic structures. *Mater. Sci. Eng. A* **639**, 208-218 (2015).
36. Woo W., Kim Y., Chae H., *et al.* Competitive strengthening between dislocation slip and twinning in cast-wrought and additively manufactured CrCoNi medium entropy alloys. *Acta Mater.* 246, 118699 (2023).
37. Xu B., Duan H., Chen X., *et al.* Harnessing instability for work hardening in multi-principal element alloys. *Nat. Mater.* **23**, 755-761 (2024).
38. Liang Z. Y., Li Y. Z. & Huang M. X. The respective hardening contributions of dislocations and twins to the flow stress of a twinning-induced plasticity steel. *Scripta Mater.* **112**, 28-31 (2016).
39. Jóni, B., Schafler, E., Zehetbauer, M., *et al.* Correlation between the microstructure studied by X-ray line profile analysis and the strength of high-pressure-torsion processed Nb and Ta. *Acta Mater.* **61**, 632-642, (2013).
40. Cao P. Y., Wang J., Jiang P., *et al.* Prediction of chemical short-range order in high-/medium-entropy alloys. *J. Mater. Sci. Technol.* **169**, 115-123 (2024).
41. Zhu S., Yan D., Zhang Y., *et al.* Strong and ductile Resinvar alloys with temperature- and time-independent resistivity. *Nat. Commun.* **15**, 7199 (2024).
42. Linus P. Atomic Radii and Interatomic Distances in Metals. *J. Am. Chem. Soc.* **69**(3), 542-553 (1947).

43. Greenwood N., Earnshaw A. Chemistry of the Elements (2nd ed.). Butterworth-Heinemann. 27–28. (1997).
44. Allred A. L. Electronegativity values from thermochemical data. Journal of Inorganic and Nuclear Chemistry. **17**(3-4), 215-221 (1961).
45. Lide D.R., (Ed.) in Chemical Rubber Company handbook of chemistry and physics, CRC Press, Boca Raton, Florida, USA, 79th edition (1998).
46. Takeuchi A., Inoue A. Classification of bulk metallic glasses by atomic size difference, heat of mixing and period of constituent elements and Its application to characterization of the main alloying element. *Mater. Trans*. **46**, 2817-2829 (2005).
47. Fantin A., Lepore G., Manzoni A., *et al*. Short-range chemical order and local lattice distortion in a compositionally complex alloy. *Acta Mater.* **193**, 329-337 (2020).
48. Fernández-Caballero A., Wróbel J., Mummery P., *et al*. Short-Range Order in High Entropy Alloys: Theoretical Formulation and Application to Mo-Nb-Ta-V-W System. *J. Phase Equilib. Diff.* **38**(4), 391-403 (2017).
49. Singh P., Smirnov A., Johnson D. Atomic short-range order and incipient long-range order in high-entropy alloys. *Phys. Rev. B* **91**, 224204 (2015).
50. Niu C., Zaddach A., Oni A., *et al*. Spin-driven ordering of Cr in the equiatomic high entropy alloy NiFeCrCo. *Appl. Phys. Lett.* **106**(16) (2015).
51. Tamm A., Aabloo A., Klintenberg M., *et al*. Atomic-scale properties of Ni-based FCC ternary, and quaternary alloys. *Acta Mater.* **99**, 307-312 (2015).
52. Liu J. P., Guo X. X., Lin Q. Y., *et al*. Excellent ductility and serration feature of metastable CoCrFeNi high-entropy alloy at extremely low temperatures. *Sci. China. Mater.* **62**(6), 853-863 (2019).
53. Han B. L., Zhang C. C., Feng K., *et al*. Additively manufactured high strength and ductility CrCoNi medium entropy alloy with hierarchical microstructure. *Mater. Sci. Eng. A* **820**, 141545 (2021).
54. Liu X. R., Feng H., Wang J., *et al*. Mechanical property comparisons between CrCoNi medium-entropy alloy and 316 stainless steels. *J. Mater. Sci. Technol.* **108**, 256-269 (2022).
55. Gludovatz B., Hohenwarter A., Thurston K. V., *et al*. Exceptional damage-tolerance of a medium-entropy alloy CrCoNi at cryogenic temperatures. *Nat. Commun*. **7**(1), 10602 (2016).
56. Zhang D. D., Zhang J. Y., Kuang J., *et al*. Superior strength-ductility synergy and strain hardenability of Al/Ta co-doped NiCoCr twinned medium entropy alloy for cryogenic applications. *Acta Mater.* **220**, 117288 (2021).
57. Ming K. S., Li B., Bai L. C., *et al*. Dynamically reversible shear transformations in a CrMnFeCoNi high-entropy alloy at cryogenic temperature. *Acta Mater.* **232**, 117937 (2022).
58. Li W. P., Chou T. H., Yang T., *et al*. Design of ultrastrong but ductile medium-entropy alloy with controlled precipitations and heterogeneous grain structures. *Appl. Mater. Today* **23**, 101037 (2021).
59. Górecki K., Bała P., Bednarczyk W., *et al*. Cryogenic behaviour of the $Al_5Ti_5Co_{35}Ni_{35}Fe_{20}$ multi-principal component alloy. *Mater. Sci. Eng. A* **745**, 346-352 (2019).
60. Zhang Z. H., Wang W., Qin S., *et al*. Dual heterogeneous structured medium-entropy alloys showing a superior strength-ductility synergy at cryogenic temperature. *J. Mater. Sci. Technol.* **17**, 3262-3276 (2022).
61. Li D. Y., Li C. X., Feng T., *et al*. High-entropy $Al_{0.3}$CoCrFeNi alloy fibers with high tensile strength and ductility at ambient and cryogenic temperatures. *Acta Mater.* **123**, 285-294 (2017).
62. Park J. M., Moon J., Bae J. W., *et al*. Role of BCC phase on tensile behavior of dual-phase Al0.5CoCrFeMnNi high-entropy alloy at cryogenic temperature. *Mater. Sci. Eng. A* **746**, 443-447 (2019).

63. Lu Y. P., Gao X. Z., Jiang L., *et al*. Directly cast bulk eutectic and near-eutectic high entropy alloys with balanced strength and ductility in a wide temperature range. *Acta Mater.* **124**, 143-150 (2017).
64. Bae J. W., Seol J. B., Moon J., *et al*. Exceptional phase-transformation strengthening of ferrous medium-entropy alloys at cryogenic temperatures. *Acta Mater.* **161**, 388-399 (2018).
65. He Z. F., Jia N., Wang H. W., *et al*. Synergy effect of multi-strengthening mechanisms in FeMnCoCrN HEA at cryogenic temperature. *J. Mater. Sci. Technol.* **86**, 158-170 (2021).
66. Haftlang F., Asghari-Rad P., Moon J., *et al*. Simultaneous effects of deformation-induced plasticity and precipitation hardening in metastable non-equiatomic FeNiCoMnTiSi ferrous medium-entropy alloy at room and liquid nitrogen temperatures. *Scripta Mater.* **202**, 114013 (2021).
67. Kwon H., Moon J., Bae J. W., *et al*. Precipitation-driven metastability engineering of carbon-doped CoCrFeNiMo medium-entropy alloys at cryogenic temperature. *Scripta Mater*. **188**, 140-145 (2020).
68. Kim D. G., Jo Y. H., Yang J., *et al*. Ultrastrong duplex high-entropy alloy with 2 GPa cryogenic strength enabled by an accelerated martensitic transformation. *Scripta Mater*. **171**, 67-72 (2019).
69. Tang K., Wu Y. K., Wei R., *et al*. Achieving superior cryogenic tensile properties in a Ti-doped $(Fe_{40}Mn_{40}Co_{10}Cr_{10})_{96.7}C_{3.3}$ high-entropy alloy by recovering deformation twinning. *Mater. Sci. Eng. A* **808**, 140927 (2021).
70. Kwon H., Sathiyamoorthi P., Karthik G. M., *et al*. 2.3 GPa cryogenic strength through thermal-induced and deformation-induced body-centered cubic martensite in a novel ferrous medium entropy alloy. *Scripta Mater.* **204**, 114157 (2021).
71. Sohn S. S., Hong S., Lee J., *et al*. Effects of Mn and Al contents on cryogenic-temperature tensile and Charpy impact properties in four austenitic high-Mn steels. *Acta Mater.* **100**, 39-52 (2015).
72. Li C. S., Li K., Dong J. B., *et al*. Mechanical behaviour and microstructure of Fe-20/27Mn-4Al-0.3C low magnetic steel at room and cryogenic temperatures. *Mater. Sci. Eng. A* **809**, 140998 (2021).
73. Anoop C. R., Singh R. K., Kumar R. R., *et al*. A Review on Steels for Cryogenic Applications. *Mater. Perform. Charact*. **10**(2), 20200193 (2021).
74. Kwon K. H., Yi I. C., Ha Y., *et al*. Origin of intergranular fracture in martensitic 8Mn steel at cryogenic temperatures. *Scripta Mater*. **69**(5), 420-423 (2013).
75. Park W. S., Chun M. S., Han M. S., *et al*. Comparative study on mechanical behavior of low temperature application materials for ships and offshore structures: Part I—Experimental investigations. *Mater. Sci. Eng. A* **528**(18), 5790-5803 (2011).
76. Luo Q., Wang H. H., Li G. Q., *et al*. On mechanical properties of novel high-Mn cryogenic steel in terms of SFE and microstructural evolution. *Mater. Sci. Eng. A* **753**, 91-98 (2019).
77. Elkot M. N., Sun B., Zhou X., *et al*. Hydrogen-assisted decohesion associated with nanosized grain boundary κ-carbides in a high-Mn lightweight steel. *Acta Mater.* **241**, 118392 (2022).
78. Wang S., Wu M. X., Shu, D., *et al*. Mechanical instability and tensile properties of TiZrHfNbTa high entropy alloy at cryogenic temperatures. *Acta Mater.* **201**, 517-527, (2020).
79. Tang L., Yan K., Cai B., *et al*. Deformation mechanisms of $FeCoCrNiMo_{0.2}$ high entropy alloy at 77 and 15 K. *Scripta Mater.* **178**, 166-170 (2020).
80. Cheng W. J., Liu W., Fan X. B., *et al*. Cooperative enhancements in ductility and strain hardening of a solution-treated Al-Cu-Mn alloy at cryogenic temperature. *Mater. Sci. Eng. A* **790**, 139707 (2020).
81. Zhemchuzhnikova D., Mogucheva A., Kaibyshev R. Mechanical properties and fracture behavior of an Al-Mg-Sc-Zr alloy at ambient and subzero temperatures. *Mater. Sci. Eng. A* **565**, 132-141 (2013).
82. Nayan N., Narayana Murty S. V., Jha A. K., *et al*. Mechanical properties of aluminium-copper-lithium alloy AA2195 at cryogenic temperatures. *Mater. Des.* **58**, 445-450 (2014).

83. Xi R., Xie J. & Yan J.-B. Evaluations of low-temperature mechanical properties and full-range constitutive models of AA 5083-H112/6061-T6. *Constr. Build. Mater.* **411**, 134520 (2024).
84. Jaladurgam N. R., Kabra S., Colliander M. H. Macro- and micro-mechanical behaviour of a γ′ strengthened Ni-based superalloy at cryogenic temperatures. *Mater. Des.* **209**, 109954 (2021).
85. Ding Q., Bei H., Wei X., *et al.* Nano-twin-induced exceptionally superior cryogenic mechanical properties of a Ni-based GH3536 (Hastelloy X) superalloy. *Mater. Today Nano,* **14**, 100110 (2021).
86. Nordström J., Siriki R., Calmunger M., *et al.* TWIP and Fracture Behavior in the Superalloy 625 at Room and Cryogenic Temperatures. *Procedia Structural Integrity* **23**, 457-462 (2019).
87. Yoshinori O., Tetsumi Y., Hideshi S. High-Cycle Fatigue Properties at Cryogenic Temperatures in INCONEL 718 Nickel-based Superalloy. *Mater. Trans.* **45**(2), 342-345 (2004).
88. Zang M. C., Niu H. Z., Yu J. S., *et al.* Cryogenic tensile properties and deformation behavior of a fine-grained near alpha titanium alloy with an equiaxed microstructure. *Mater. Sci. Eng. A* **840**, 142952 (2022).
89. Zang M. C., Niu H. Z., Zhang H. R., *et al.* Cryogenic tensile properties and deformation behavior of a superhigh strength metastable beta titanium alloy Ti-15Mo-2Al. *Mater. Sci. Eng. A* **817**, 141344 (2021).
90. Zang, M. C., Niu, H. Z., Liu, S., Guo, R. Q. & Zhang, D. L. Achieving highly promising strength-ductility synergy of powder bed fusion additively manufactured titanium alloy components at ultra-low temperatures. *Addit. Manuf.* **65**, 103444 (2023).
91. Zhao W., Su W., Li L., *et al.* Evolution of mechanical properties of Ti-6Al-4V alloy in the temperature range of 20 to -196 °C. *Met. Mater. Int.* **27**(9), 3214-3224 (2021).
92. Kumar P., Michalek M., Cook D., *et al.* On the strength and fracture toughness of an additive manufactured CrCoNi medium-entropy alloy. *Acta Mater.* **258**, 119249 (2023).
93. Jo Y., Doh K., Kim D., *et al.* Cryogenic-temperature fracture toughness analysis of non-equi-atomic $V_{10}Cr_{10}Fe_{45}Co_{20}Ni_{15}$ high-entropy alloy. *J. Alloy. Comp.* **809**, 151864 (2019).
94. Yang, J. *et al.* Effects of deformation-induced martensitic transformation on cryogenic fracture toughness for metastable Si8V2Fe45Cr10Mn5Co30 high-entropy alloy. *Acta Mater.* **225**, 117568 (2022).
95. Strife J., Passoja D. The effect of heat treatment on microstructure and cryogenic fracture properties in 5Ni and 9Ni steel. *Metal. Trans. A* **11**(8), 1341-1350 (1980).
96. Ishizaka J., Shimamoto R., Nakajima H. Strength and toughness of 12Cr-12Ni-10Mn-5Mo steel for cryogenic structural application. *Tetsu-to-Hagane*, **76**(5), 791-798 (1990).
97. Xiao Y., Qian G., Sun J., *et al.* Effects of temperature on tensile and fracture performance of Ti6Al4V alloy fabricated by laser powder bed fusion. *Theor. Appl. Fract. Mec.* **125**, 103931 (2023).
98. Nagai K., Yuri T., Ogata T., Umezawa O., Ishikawa K., Nishimura T., Mizoguchi T., Ito Y. Cryogenic mechanical properties of Ti-6Al-4V alloys with three levels of oxygen content. *ISIJ Int.* **31**(8), 882-889 (1991).
99. Nagai K., Ishikawa K., Mizoguchi T., Ito Y. Strength and fracture toughness of Ti5Al2.5Sn ELI alloy at cryogenic temperatures. *Cryogenics* **26**(1), 19-23 (1986).
100. Semenova I., Modina J., Polyakov A., *et al.* Fracture toughness at cryogenic temperatures of ultrafine-grained Ti-6Al-4V alloy processed by ECAP. *Mater. Sci. Eng. A* **716**, 260-267 (2018).
101. Rao K., Ritchie R. Mechanisms influencing the cryogenic fracture-toughness behavior of aluminum-lithium alloys. *Acta metallurgica et materialia*, **38**(11), 2309-2326 (1990).
